# Supplementary material for: Injury profile suffered by targets of antipersonnel improvised explosive devices: prospective cohort study
Source: BMJ Open. 2017 Aug 7;7(7):e014697. doi: 10.1136/bmjopen-2016-014697 (PMC5691184; doi:10.1136/bmjopen-2016-014697)
Supplement: Author's manuscript [file bmjopen-2016-014697.draft_revisions.pdf]

BMJ Open is committed to open peer review. As part of this commitment we make the peer review history of every article we publish publicly available.

When an article is published we post the peer reviewers' comments and the authors' responses online. We also post the versions of the paper that were used during peer review. These are the versions that the peer review comments apply to.

The versions of the paper that follow are the versions that were submitted during the peer review process. They are not the versions of record or the final published versions. They should not be cited or distributed as the published version of this manuscript.

BMJ Open is an open access journal and the full, final, typeset and author-corrected version of record of the manuscript is available on our site with no access controls, subscription charges or pay-per-view fees (http://bmjopen.bmj.com).

If you have any questions on BMJ Open's open peer review process please email editorial.bmjopen@bmj.com

BMJ Open

Profile of injuries suffered by the target of an antipersonnel improvised explosive device

Journal:	*BMJ Open*
Manuscript ID	bmjopen-2016-014697
Article Type:	Research
Date Submitted by the Author:	03-Nov-2016

Complete List of Authors:	Smith, Shane; Royal Canadian Medical Service; Western University, Surgery<br>Devine, Melissa ; Royal Canadian Medical Service, Critical Care Nursing<br>Taddeo, Joseph; Maine Veterans' Affairs Medical Center, Surgery<br>McAlister, Vivian; Royal Canadian Medical Service, ; Western University, Surgery

**Primary Subject Heading**:	Global health
Secondary Subject Heading:	Emergency medicine, Surgery
Keywords:	blast injury, war, amputation, soft tissue injury, landmines

SCHOLARONE™ Manuscripts

Injury profile suffered by targets of the antipersonnel improvised explosive device

Shane Smith[1,2]

Melissa Devine[2]

Joseph Taddeo[3]

Vivian McAlister[1,2*]

[1]Royal Canadian Medical Service

[2]Division of General Surgery, University of Western Ontario, Ontario, Canada

[3]Maine Veterans' Affairs Medical Center, Augusta, Maryland, USA

Shane Smith

Medical Officer, Royal Canadian Medical Service

C4-211 University Hospital, London, Ontario, Canada, N5X 2S1

Melissa Devine

Critical Care Nursing Officer, Royal Canadian Medical Service

QEII Health Science Centre, 1276 South Park Street 3rd Floor Bethune, Room 371

Halifax, NS B3H 2Y9

Joseph Taddeo

General-Trauma Surgeon, Maine Veterans' Affairs Medical Center

1 VA Center Augusta, ME 04330, USA

For peer review only - http://bmjopen.bmj.com/site/about/guidelines.xhtml

Vivian McAlister

General Surgeon, Royal Canadian Medial Service

C8-005 University Hospital, London, Ontario, Canada, N5X 2S1.

Telephone: (519) 663-2920, Fax: 1-(855)-538-2926

Email: *vmcalist@uwo.ca*

***Corresponding author**

Word count: 2,506

Keywords: Improvised Explosive Device; IED; Blast; Amputation; Trauma; Military;

For peer review only

For peer review only - http://bmjopen.bmj.com/site/about/guidelines.xhtml

The views expressed in this paper are those of the authors and do not constitute the views or policies of the Canadian Armed Forces or the United States Navy.

Competing interests: All authors have completed the ICMJE uniform disclosure form at www.icmje.org/coi_disclosure.pdf and declare: no support from any organisation for the submitted work; no financial relationships with any organisations that might have an interest in the submitted work in the previous three years; no other relationships or activities that could appear to have influenced the submitted work.

Contributions: S.S. analysed the data and drafted the manuscript. M.D. conducted data collection. V.M. and J.T. were the treating trauma surgeons of the casualties and designers of the study. They also conducted data collection. The manuscript has been reviewed and approved by all authors. All authors had full access to all of the data in the study and can take responsibility for the integrity of the data and the accuracy of the data analysis.

This study did not receive any outside funding. The Canadian Armed Forces employed S.S., M.D., and V.M. at the time of the study. The United States Navy employed J.D. at the time of the study.

Transparency declaration: The lead author affirms that the manuscript is an honest, accurate, and transparent account of the study being reported; that no important aspects of the study have been omitted; and that any discrepancies from the study as planned have been explained.

Data sharing: no additional data available.

For peer review only - http://bmjopen.bmj.com/site/about/guidelines.xhtml

ABSTRACT

Objective: To describe the injury pattern caused by the antipersonnel improvised explosive device (AP-IED) upon its target.

Design: The injuries of sequential patients, targeted by an AP-IED, were prospectively recorded and described. The target was defined as a casualty of an AP-IED who received an amputation.

Setting: Role 3 Multinational Medical Unit in Kandahar Air Field, Afghanistan.

Participants: Consecutive patients who met the definition of being an AP-IED target, without regard for gender, nationality or military status, which eventually included 100 males between the ages of 6 and 44 years of age.

Main outcome measures: The details of injuries were recorded in order to describe the pattern and characterize the injuries suffered by the target of AP-IEDs. The level of amputation, the level of soft tissue injury, the fracture pattern (including pelvic fractures) as well as perineal, gluteal, genital, and other injuries were recorded.

Results: Information was collected on a cohort of 100 consecutive victims of AP-IEDs who experienced limb amputations. Multiple amputations occured in 70 patients: 5 quadruple amputations, 27 triple amputations, and 38 double amputations. Pelvic fracture occurred in 21 victims, all but one of whom had multiple amputations. Severe perineal, gluteal, or genital injuries were present in 46 patients. Severe soft tissue injury was universal, with injection of contaminated soil along tissue planes well above entry sites. There were 13 facial injuries, 9 skull fractures, and 3 traumatic brain injuries. Eleven eye injuries were seen; none of the victims with eye injuries were wearing eye protection.

For peer review only - http://bmjopen.bmj.com/site/about/guidelines.xhtml

For peer review only

The casualty fatality rate was at least 19%.

Conclusion: The injury pattern suffered by the target of the AP-IED consists of multiple extremity amputations, pelvic ring disruption, and severe soft tissue injury extending into gluteal muscles, perineum and genitals, with deep tissue contamination by soil. This injury profile is markedly worse than that of antipersonnel landmines.

For peer review only

For peer review only - http://bmjopen.bmj.com/site/about/guidelines.xhtml

ARTICLE SUMMARY

Strengths and limitations of this study:

This is a prospective consecutive series with a predefined definition of the target of the AP-IED attack used as inclusion criteria.

The definition of the target of the AP-IED was chosen so as to facilitate comparison to the International Committee of the Red Cross land mine data. This excluded injured bystanders who may have more minor injuries.

The Role 3 Multinational Medical Unit was the only surgical facility in the region. This maximized catchment of AP-IED victims and minimized selection bias.

Patients who died at the scene of the explosion may not have been evacuated to the Role 3 Multinational Medical Unit and would not have been included in this study. This may have resulted in an underestimation of the AP-IED's lethality.

For peer review only

For peer review only - http://bmjopen.bmj.com/site/about/guidelines.xhtml

INTRODUCTION

The improvised explosive device (IED) is increasingly used in modern conflicts. In the recent Afghan war, fewer coalition soldiers were injured with rifles and ballistic weapons than with IEDs [1,2]. IEDs tend to be classified in defense literature by the type of explosive or delivery system (for example suicide or car bomb). Medically, it is more appropriate to classify IEDs by their target: crowd attack IED (CA-IED); anti-vehicle IED (AV-IED) and the anti-personnel IED (AP-IED). CA-IEDs are designed to injure groups collected in small areas. Increasingly CA-IEDs are deployed against soft undefended targets as measures to protect military and institutional targets improve. In recent conflicts, the use of AV-IEDs, large weapons that require time and effort to position, was reduced by improved intelligence and surveillance. They was replaced by AP-IEDs, smaller devices designed to target an individual, usually on foot [3-6]. Different injury patterns might be expected from each type of IED. AV-IEDs cause injuries similar to high-energy motor vehicle collisions. Injuries from CA-IEDs are caused by fragments, some of which may contain human tissue, and may be magnified by enclosing walls. The purpose of this paper is to describe the pattern of injury suffered by the target of the AP-IED, whose body may absorb a large amount of energy directly.

For peer review only - http://bmjopen.bmj.com/site/about/guidelines.xhtml

For peer review only

Antipersonnel landmines came in to widespread use in the Second World War. They were designed to injure but not kill, in order to remove a target from combat and to increase the logistical burden of caring for the casualty. These weapons are typically buried and left for the target to trigger. The weapon is indiscriminate because non-combatants such as children and civilians may detonate them. They are also frequently left buried and active after fighting in that region, or the conflict itself, has ended. Because of this indiscriminate nature, 162 countries agreed to stop the production and use of landmines by signing the Ottawa Treaty in 1999[7].

The injury pattern from antipersonnel mines was characterized by Coupland and Korver for the International Committee of the Red Cross (ICRC) [8, 9]. In pattern 1 injuries, the victim triggers the mine and suffers the effect of the explosion. In pattern 2 injuries, bystanders suffer fragment wounds when the device is triggered. In pattern 3 injuries, the mine explodes while being handled or played with by children, resulting in injuries to the hands and face. Their examination of 4616 antipersonnel mine victims presenting to ICRC hospitals over 5 years found that 1077 required amputation; 606 required unilateral below the knee amputation (BKA) (56.3%), 26 bilateral BKA (2.4%), 349 unilateral above the knee amputations (AKA) (32.4%), 57 bilateral AKA (5.3%), and 39 a combination of AKA and BKA (3.6%). Their description of the injury profile suffered by the target who triggered an antipersonnel mine, which has been confirmed by other studies, is of a traumatic amputation of the foot or leg with less severe injuries elsewhere [8-11]. Pattern 2 injuries included penetration by fragments but did not involve traumatic amputation. Therefore traumatic amputation has been used to distinguish targets of

For peer review only - http://bmjopen.bmj.com/site/about/guidelines.xhtml

landmines from bystanders[8, 9]. The objective of this study was to describe the pattern of injury caused by the AP-IED.

METHODS

The target of an AP-IED was defined as the pedestrian victim of an IED with a traumatic amputation. In order to be included in the study, casualties were required to have been reported as being the dismounted (not in a vehicle) victim of an IED explosion and to have suffered a traumatic amputation. Casualties of either the AV-IED or CA-IED were excluded. The study was designed to describe the injuries suffered by 100 consecutive targets of AP-IED attacks who presented to the North Atlantic Treaty Organization Role 3 Multinational Medical Unit (R3-MMU) in Kandahar Air Field (KAF). The hospital received casualties from the point of injury or via a forward treatment centre[12]. A R3-MMU is the highest level of in-theatre medical care and would be equivalent to a civilian level-II trauma centre, as defined by the American College of Surgeons-Committee On Trauma. A specifically designated trauma nurse (M.D.), and attending surgeons (J.T., V.M.) prospectively collected data for each patient. Data was collected between January 2010 and July 2011, or until data from 100 victims had been obtained. Data collected included: nationality, age, gender, mortality status, specific injuries, and imaging results. The level of traumatic amputation, the level of soft tissue injury, the fracture pattern (including pelvic fractures) as well as perineal and gluteal injuries were specifically recorded. Generally, coalition patients were transferred after damage control surgery, whereas local patients also received definitive care at the R3-MMU. Data were collected until the time of first discharge from the hospital. In order to minimize bias patients

For peer review only - http://bmjopen.bmj.com/site/about/guidelines.xhtml

included sequentially as a consecutive series with a predefined definition of AP-IED target. The R3-MMU was the only surgical facility in the region and would receive all AP-IED victims for care. A limitation may be that some victims killed at the scene of the explosion may not have been evacuated to the R3-MMU resulting in an underestimation of the casualty fatality rate. Patient level data is reported identifying the injuries of each of the 100 victims. The paediatric victims are also presented separately. The data collection was complete with injury data captured for all 100 victims. This study was approved by the commander of the R3-MMU KAF and by the Research Ethics Board of the University of Western Ontario (REB # 104124).

RESULTS

One hundred consecutive casualties with amputations from AP-IEDs were identified and their injuries were described. All of the patients were male. The mean, and median, age of the victims was 25 years. The age range was from ages 6 to 44 years. There were 9 patients under the age of 18 (Figure 1). Twenty-seven patients were Afghan local nationals and 61 were coalition soldiers (USA, Canada, UK); there were 12 victims whose nationality was not captured. Eleven patients were dead upon arrival and another 8 died of their wounds in the hospital, giving a 19% casualty fatality rate. Gender, injury pattern, age or approximate age, was recorded for all victims. There were 12 victims whose nationality was not specified (Table 1).

Seventy victims (70%) sustained multiple amputations: 5 patients suffered quadruple amputations, 27 had triple amputations, and 38 victims had double amputations (Figure

For peer review only - http://bmjopen.bmj.com/site/about/guidelines.xhtml

2). Two victims had hip disarticulations, 41 had at least one AKA, and 56 had at least one BKA (including thought knee and through ankle amputations). Sixty-five victims had bilateral lower extremity amputations and 37 had at least one upper extremity amputation (at the level of the hand or higher) (Table 2). Pelvic fractures were present in 21 victims, all but one of whom had multiple amputations. Forty-six patients had perineal and gluteal injuries; these included 8 anorectal injuries, 8 penile injuries, and 26 scrotal injuries including 10 orchiectomies. There were 13 facial injuries, 9 skull fractures and 3 traumatic brain injuries. Eye injuries were found in 11 patients, none of whom were wearing eye protection. Of the 9 pediatric patients, 3 had triple amputations and 5 had double amputations. There was one pelvic fracture and two perineal injuries among the pediatric victims (Table 3).

Therefore, the typical injury profile suffered by targeted victims of AP-IEDs included: bilateral lower extremity amputations (often above the knee); frequent upper extremity amputations; extensive soft tissue injury with deep contamination by soil, extending into gluteal and perineal regions; pelvic ring disruption and genital mutilation.

DISCUSSION

The AP-IED has been likened to an improvised antipersonnel landmine. There is an implication that it is a primitive or crude weapon crafted from locally available resources because of a lack of access to conventional weapons. In fact, it is a much more destructive weapon than the conventional antipersonnel landmine. We used the ICRC landmine injury classification system to define the target of either the landmine or the AP-IED. Whereas antipersonnel mines typically result in a unilateral BKA, the AP-IED causes

For peer review only - http://bmjopen.bmj.com/site/about/guidelines.xhtml

For peer review only

bilateral high amputation of the lower extremities. Triple amputations are not seen with landmines but occurred in 27% of victims of AP-IEDs. Severe perineal and gluteal injury with soft tissue contamination with soil was commonly present in the victims of the AP-IED but is not seen among landmine injuries. The force endured by targeted victims of AP-IEDs must be far greater than that caused by landmines. A measure of that force is the fact that it sufficiently powerful to disrupt the pelvic ring in one fifth of the patients (Figure 3).

It is not the purpose of this paper to undermine the severity of injury caused by landmines, which are often devastating, ruining lives. Both antipersonnel landmines and AP-IEDs injure indiscriminately. In this series, almost one in ten victims of AP-IEDs was a child. The AP-IED has this tragic feature in common with landmines. Even more disconcerting is the frequency with which children suffer severe injuries from the powerful explosive force of the AP-IED: 89% suffered multiple amputations and 33% lost three limbs.

The AP-IED victims wore different types of personal protective equipment (PPE). Coalition soldiers wore PPE including helmets, body armour, and ballistic eyewear. Each nation issued its own style and brand of PPE. Local Afghan soldiers often used loose-fitting helmets but rarely had body armour and almost never used ballistic eyewear. Civilian casualties were not wearing any PPE. The effect of PPE was apparent, as thoracoabdominal injuries and eye injuries did not occur in victims wearing body armour or ballistic eye wear respectively. The severe perineal soft tissue injuries caused by AP-IEDs might also be mitigated by specialized PPE for the perineum that is currently being

For peer review only - http://bmjopen.bmj.com/site/about/guidelines.xhtml

For peer review only

developed. It can be can hypothesized that soldiers on patrol, holding their weapon in a low ready position with a flexed elbow, may benefit from PPE designed to protect the elbow.

It is possible that we have magnified the severity of the injury pattern by concentrating our focus upon patients that met our definition of being targeted by the AP-IED, that is having sustained at least one limb amputation. However our findings are in keeping with other descriptions of IED injuries in the literature. Jacobs *et al*. classified the limb injury pattern of 103 consecutive casualties of IEDs treated at a UK Role 3 facility in Helmand Province, Afghanistan. They included victims who did not have amputation of a limb. They found 76 victims suffered significant bilateral lower limb injuries, with 50 who required bilateral lower limb amputation. Thirty-three victims suffered genital or perineal injury, 9 sustained pelvic ring disruption, and 40 sustained significant upper limb injury. They found that all pelvic fractures and 80% of genital injuries were sustained among casualties with bilateral lower limb amputations. [5] A retrospective review of the UK Joint Theatre Trauma Registry examined UK services personnel who were casualties of IEDs in Afghanistan, who sustained lower extremity amputations between January 2007 and December 2010 [4]. This registry includes post-mortem reports of soldiers who were killed and never received hospital care. The registry is restricted to UK services members and contains no local nationals or civilian injuries. They examined 656 IED victims with 138 killed in action (21%) and 31 who died of their wounds (4.7%) resulting in a 25.9% casualty fatality rate. Of the 169 victims who sustained a traumatic lower extremity amputation, 69 were killed in action (40.8%) and 31 died of their wounds (18.3%). They found that the level of amputation was inversely correlated with survival with only two

For peer review only - http://bmjopen.bmj.com/site/about/guidelines.xhtml

For peer review only

survivors from hindquarter amputation out of 39 patients with this level of injury. Bilateral above knee amputation was the point at which more patients survived to reach hospital, as demonstrated by a halving of the fatality rate.[4] Another retrospective review of the UK Military Trauma Registry focused on soldiers from Afghanistan with bilateral leg amputation. They examined 43 casualties with at least two amputations; 80% of these were from AP-IEDs with the other 20% being from AV-IEDs. The most common bilateral amputation was a bilateral AKA (58%), and 20% of victims suffered triple amputation. 14% of victims sustained open pelvic fractures and 44% suffered perineal or genital injury. No victims survived the loss of all four limbs [6].

The soft tissue injury is a particularly difficult problem encountered by those caring for victims of AP-IEDs. The focused explosion forces soil up along soft tissue planes far above the point of entry. This may worsen the level of eventual amputation and it condemns the victim to multiple operations to remove the contamination. Even still, it may leave the victim at the mercy of unusual antibiotic resistant soil organisms such as *Acinetobacter baumannii*[13].

Knowing the pattern of injury helps responders tailor care. As Jacobs *et al*. have suggested, first responders should apply tourniquets bilaterally even if the victim is not bleeding, as haemorrhage is likely to start once resuscitation restores intravascular volume[5]. While current protocols require medics to apply a neck collar to all victims of IEDs, spinal injury is rare in AP-IED victims despite the magnitude of the force[3]. On the other hand, we recommend that all AP-IED victims with perineal injury have pelvic binders placed by the first responders to reduce the large amount of blood that may be lost

For peer review only - http://bmjopen.bmj.com/site/about/guidelines.xhtml

silently with pelvic disruption. Multiple surgical teams have to work simultaneously to rescue these patients. Surgeons have to be adept at dealing with pelvic and gluteal bleeding, sometimes by ligating the internal iliac artery. Rehabilitation requires mobilization on temporary lower limb prostheses despite injured upper limbs. Finally, victims of this severe injury pattern need life-long support because disabilities may become more pronounced as the ability to compensate fades with age.

In collecting information on 100 consecutive patients we hope to have describes a generalizable result. Due to the nature of the conflict and the local culture all of the patients examined were male. While we would expect the severity of the injury pattern to be generalizable to females we have not explicitly examined the effect of AP-IED on the female pelvis and perineum.

Despite the huge force endured by the targeted victim of the AP-IED, the casualty fatality rate that we observed was only 19%. This rate is an underestimate, as some casualties who were killed at the site of the AP-IED may not have been brought to our facility. In addition, death that occurred after discharge from our facility was not recorded. Data regarding anti-personnel landmines is also affected by this problem. The observed mortality rate with AP-IED is of the same order as that reported with anti-personnel landmines[14]. This information has a disturbing implication. The military purpose of the AP-IED has not been studied. It would appear to be similar to the antipersonnel landmine: to disable but not kill an opponent. However the AP-IED, by its nature, causes superfluous injury and unnecessary suffering. Just as the reports by Coupland and Korver provided the medical evidence upon which the banning of landmines was based, it is

For peer review only - http://bmjopen.bmj.com/site/about/guidelines.xhtml

hoped that reports regarding the pattern of injury caused by the AP-IED will result in an abhorrence of this weapon and those who use it.

For peer review only

For peer review only - http://bmjopen.bmj.com/site/about/guidelines.xhtml

REFERENCES

1. Jacobs N, Taylor DM, Parker PJ. Changes in surgical workload at the JF Med Gp Role 3 Hospital, Camp Bastion, Afghanistan, November 2008-November 2010. *Injury.* 2012;43(7):1037-1040.

2. Beckett A, Pelletier P, Mamczak C, Benfield R, Elster E. Multidisciplinary trauma team care in Kandahar, Afghanistan: current injury patterns and care practices. *Injury.* 2012;43(12):2072-2077.

3. Taddeo J, Devine M, McAlister VC. Cervical spine injury in dismounted improvised explosive device trauma. *Canadian journal of surgery. Journal canadien de chirurgie.* 2015;58(3 Suppl 3):S104-107.

4. Morrison JJ, Hunt N, Midwinter M, Jansen J. Associated injuries in casualties with traumatic lower extremity amputations caused by improvised explosive devices. *The British journal of surgery.* 2012;99(3):362-366.

5. Jacobs N, Rourke K, Rutherford J, et al. Lower limb injuries caused by improvised explosive devices: proposed 'Bastion classification' and prospective validation. *Injury.* 2014;45(9):1422-1428.

6. Penn-Barwell JG, Bennett PM, Kay A, Sargeant ID. Acute bilateral leg amputation following combat injury in UK servicemen. *Injury.* 2014;45(7):1105-1110.

7. United Nations. Convention on the Prohibition of the Use, Stockpiling, Production and Transfer of Anti-Personnel Mines and on their Destruction. *United Nations, Treaty Series , vol. 2056, p. 211.* 1999.

For peer review only - http://bmjopen.bmj.com/site/about/guidelines.xhtml

8. Coupland RM, Korver A. Injuries from antipersonnel mines: the experience of the International Committee of the Red Cross. *BMJ (Clinical research ed.).* 1991;303(6816):1509-1512.

9. Korver AJ. Injuries of the lower limbs caused by antipersonnel mines: the experience of the International Committee of the Red Cross. *Injury.* 1996;27(7):477-479.

10. Coupland RM. Amputation for antipersonnel mine injuries of the leg: preservation of the tibial stump using a medial gastrocnemius myoplasty. *Annals of the Royal College of Surgeons of England.* 1989;71(6):405-408.

11. Soroush A, Falahati F, Zargar M, Soroush M, Khateri S, Khaji A. Amputations due to landmine and unexploded ordinances in post-war Iran. *Archives of Iranian medicine.* 2008;11(6):595-597.

12. Brisebois R, Hennecke P, Kao R. et al. The Role 3 Multinational Medical Unit at Kandahar Airfield 2005-2010. *Can J Surg*. 2011;54(6):S124-9.

13. O'Shea MK. Acinetobacter in modern warfare. *Int J Antimicrob Agents.* 2012;39(5):363-75.

14. Bilukha OO, Brennan M, Woodruff BA. Death and injury from landmines and unexploded ordnance in Afghanistan. *JAMA.* 2003;290(5):650-653.

For peer review only

For peer review only - http://bmjopen.bmj.com/site/about/guidelines.xhtml

Table 1: Victim characteristics

Characteristic	
Number of patients	100
Age, mean (SD)	25 (6.8) yrs
Median	25 yrs
Range	6 – 44 yrs
Male	100
Female	0
Nationality	
Afghan	27
Coalition	61
American	49
Canadian	8
UK	4
Not specified	12
Mortality	19
Killed in Action	11
Died of wounds	8

SD = standard deviation

For peer review only

For peer review only - http://bmjopen.bmj.com/site/about/guidelines.xhtml

Table 2: Distribution of amputations

Amputation Type	Overall	Pelvic Fracture	Perineal, Gluteal, Genital Injury	Killed in Action	Died of Wounds	Casualty Fatality Rate
HipDis/TKA/UEA	1	0	0	0	1	1/1
HipDis/-	1	0	0	0	1	1/1
AKA/AKA/UEA/UEA	4	0	3	0	0	0/4
AKA/AKA/UEA	7	3	6	0	0	0/7
AKA/AKA	8	2	4	2	1	3/8
AKA/BKA/UEA	2	1	0	1	1	2/2
AKA/TKA/UEA	1	1	0	0	1	1/1
AKA/BKA	7	1	5	0	0	0/7
AKA/TKA	7	4	6	0	0	0/7
AKA/UEA/UEA	1	0	0	0	0	0/1
AKA/UEA	1	0	0	0	0	0/1
AKA/TAA	1	0	0	0	0	0/1
AKA/-	2	0	0	0	0	0/2
BKA/BKA/UEA/UEA	1	0	0	0	1	1/1
BKA/BKA/UEA	13	4	6	4	2	6/13
BKA/TKA/UEA	1	0	1	0	0	0/1
BKA/BKA	9	3	5	2	0	2/9
BKA/UEA	2	0	0	0	0	0/2
BKA/TKA	1	0	0	1	0	1/1
BKA/-	22	1	9	1	0	1/22
TKA/TKA/UEA	1	1	1	0	0	0/1
TKA/TKA	1	0	0	0	0	0/1
TKA/-	1	0	0	0	0	0/1
TAA/UEA	1	0	0	0	0	0/1
TAA/-	3	0	0	0	0	0/3
UEA/-	1	0	0	0	0	0/1

HipDis, hip disarticulation; AKA, above knee amputation; BKA, below knee amputation; TKA, though knee amputation, TAA, though ankle amputation; UEA, upper extremity amputation, which includes above elbow, below elbow and hand amputations.

For peer review only

Table 3: Distribution of pediatric amputations

Age	Amputation Type	Pelvic Fracture	Perineal, Gluteal, Genital Injury	Disposition
16	AKA/BKA	No	No	Survived
14	AKA/AKA	No	Yes	DOW
14	AKA/UEA/UEA	No	No	Survived
11	UEA/TKA/AKA	Yes	No	DOW
10	BKA/BKA/UEA	No	No	KIA
10	UEA/TAA	No	No	Survived
10	BKA/AKA	No	Yes	Survived
10	BKA/BKA	No	No	KIA
6	UEA	No	No	Survived

AKA, above knee amputation; BKA, below knee amputation; TKA, though knee amputation, TAA, though ankle amputation; UEA, upper extremity amputation, which includes above elbow, below elbow and hand amputations; DOW, died of wounds; KIA, killed in action.

For peer review only

For peer review only - http://bmjopen.bmj.com/site/about/guidelines.xhtml

Figure 1: Victim ages

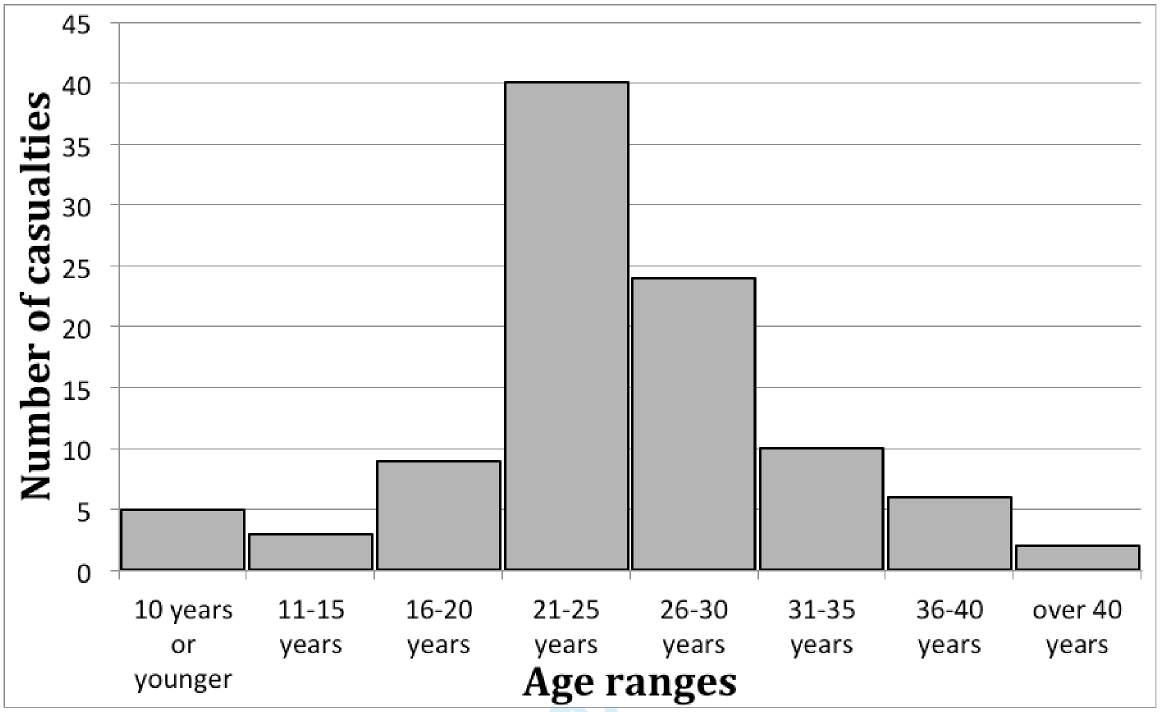

For peer review only

For peer review only - http://bmjopen.bmj.com/site/about/guidelines.xhtml

Figure 2: Number of amputations per victim

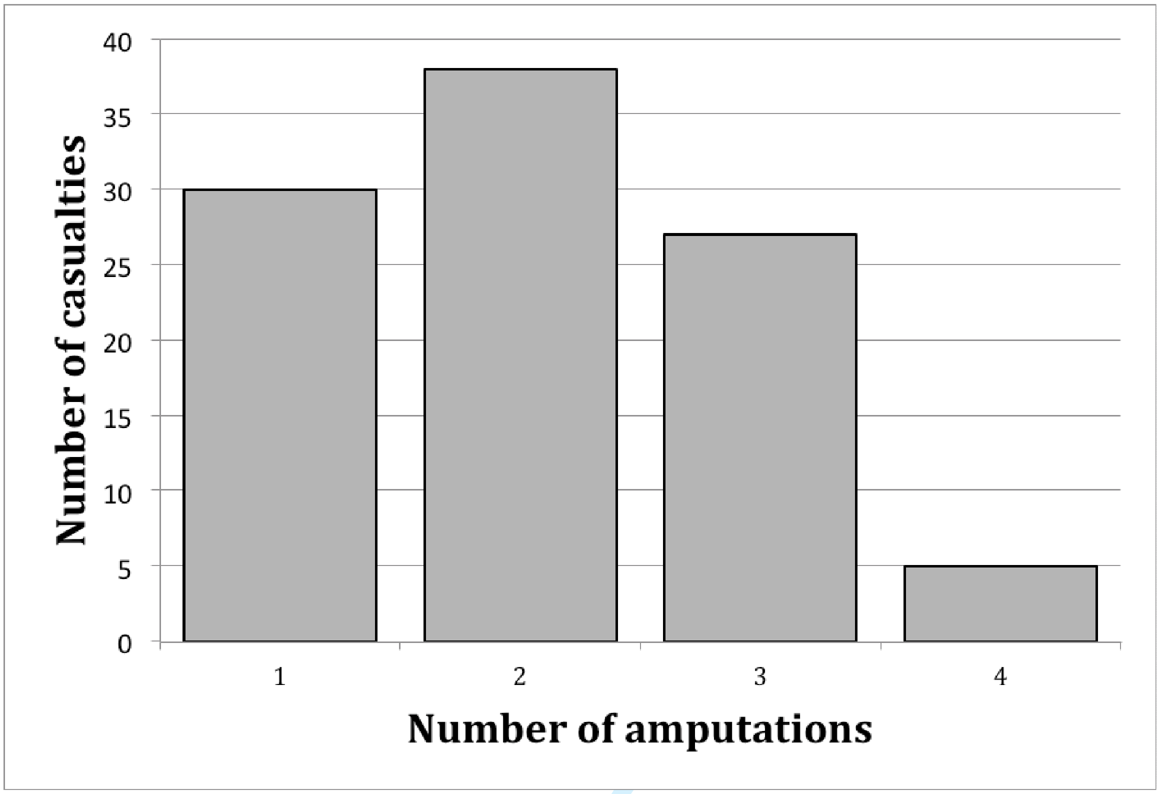

For peer review only

For peer review only - http://bmjopen.bmj.com/site/about/guidelines.xhtml

Figure 3:

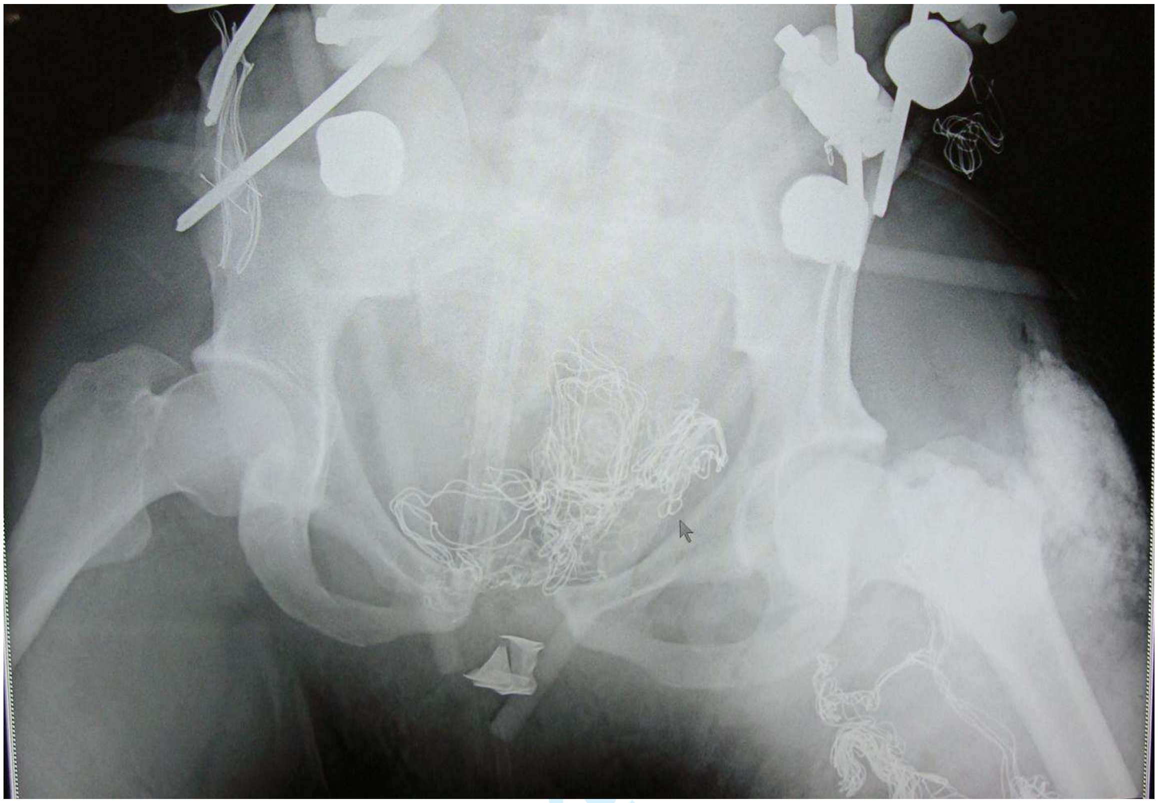

For peer review only - http://bmjopen.bmj.com/site/about/guidelines.xhtml

Legend for figures

Figure 1: Age ranges of AP-IED targeted victims

Figure 2: Numbers of traumatic limb amputations per targeted victim of the AP-IED

Figure 3: X-ray taken for placement of a pelvic external fixator showing: disruption of pelvic ring by the force of an anti-personnel improvised explosive device; the arrow indicates combat gauze packing a severe perineal injury; silica laden soil injection by the explosion.

For peer review only

For peer review only - http://bmjopen.bmj.com/site/about/guidelines.xhtml

STROBE Statement—checklist of items that should be included in reports of observational studies

	Item No	Recommendation
**Title and abstract**	1	(*a*) Indicate the study's design with a commonly used term in the title or the abstract **Pg 4**
		(*b*) Provide in the abstract an informative and balanced summary of what was done and what was found **Pg 4**
**Introduction**		
Background/rationale	2	Explain the scientific background and rationale for the investigation being reported **Pg 7-8**
Objectives	3	State specific objectives, including any prespecified hypotheses **Pg 9**
**Methods**		
Study design	4	Present key elements of study design early in the paper **Pg 9**
Setting	5	Describe the setting, locations, and relevant dates, including periods of recruitment, exposure, follow-up, and data collection **Pg 9**
Participants	6	(*a*) *Cohort study*—Give the eligibility criteria, and the sources and methods of selection of participants. Describe methods of follow-up **Pg 9**<br>*Case-control study*—Give the eligibility criteria, and the sources and methods of case ascertainment and control selection. Give the rationale for the choice of cases and controls<br>*Cross-sectional study*—Give the eligibility criteria, and the sources and methods of selection of participants
		(*b*) *Cohort study*—For matched studies, give matching criteria and number of exposed and unexposed N/A<br>*Case-control study*—For matched studies, give matching criteria and the number of controls per case
Variables	7	Clearly define all outcomes, exposures, predictors, potential confounders, and effect modifiers. Give diagnostic criteria, if applicable **Pg 9**
Data sources/ measurement	8*	For each variable of interest, give sources of data and details of methods of assessment (measurement). Describe comparability of assessment methods if there is more than one group **Pg 9**
Bias	9	Describe any efforts to address potential sources of bias **Pg 10**
Study size	10	Explain how the study size was arrived at **Pg 9**
Quantitative variables	11	Explain how quantitative variables were handled in the analyses. If applicable, describe which groupings were chosen and why **N/A**
Statistical methods	12	(*a*) Describe all statistical methods, including those used to control for confounding **Pg 10**
		(*b*) Describe any methods used to examine subgroups and interactions **Pg 10**
		(*c*) Explain how missing data were addressed **Pg 10**
		(*d*) *Cohort study*—If applicable, explain how loss to follow-up was addressed<br>*Case-control study*—If applicable, explain how matching of cases and controls was addressed<br>*Cross-sectional study*—If applicable, describe analytical methods taking account of sampling strategy
		(*e*) Describe any sensitivity analyses

Continued on next page

For peer review only - http://bmjopen.bmj.com/site/about/guidelines.xhtml

**Results**		
Participants	13*	(a) Report numbers of individuals at each stage of study—eg numbers potentially eligible, examined for eligibility, confirmed eligible, included in the study, completing follow-up, and analysed **Pg 10**
		(b) Give reasons for non-participation at each stage
		(c) Consider use of a flow diagram
Descriptive data	14*	(a) Give characteristics of study participants (eg demographic, clinical, social) and information on exposures and potential confounders **Pg 10**
		(b) Indicate number of participants with missing data for each variable of interest **Pg 10 – Table 1**
		(c) *Cohort study*—Summarise follow-up time (eg, average and total amount)
Outcome data	15*	*Cohort study*—Report numbers of outcome events or summary measures over time
		Case-control study—Report numbers in each exposure category, or summary measures of exposure
		Cross-sectional study—Report numbers of outcome events or summary measures
Main results	16	(*a*) Give unadjusted estimates and, if applicable, confounder-adjusted estimates and their precision (eg, 95% confidence interval). Make clear which confounders were adjusted for and why they were included **Pg 10-11**
		(*b*) Report category boundaries when continuous variables were categorized
		(*c*) If relevant, consider translating estimates of relative risk into absolute risk for a meaningful time period
Other analyses	17	Report other analyses done—eg analyses of subgroups and interactions, and sensitivity analyses
**Discussion**		
Key results	18	Summarise key results with reference to study objectives **Pg 12**
Limitations	19	Discuss limitations of the study, taking into account sources of potential bias or imprecision. Discuss both direction and magnitude of any potential bias **Pg 12**
Interpretation	20	Give a cautious overall interpretation of results considering objectives, limitations, multiplicity of analyses, results from similar studies, and other relevant evidence **Pg 13-14**
Generalisability	21	Discuss the generalisability (external validity) of the study results **Pg 15**
**Other information**		
Funding	22	Give the source of funding and the role of the funders for the present study and, if applicable, for the original study on which the present article is based **Pg 3**

*Give information separately for cases and controls in case-control studies and, if applicable, for exposed and unexposed groups in cohort and cross-sectional studies.

Note: An Explanation and Elaboration article discusses each checklist item and gives methodological background and published examples of transparent reporting. The STROBE checklist is best used in conjunction with this article (freely available on the Web sites of PLoS Medicine at http://www.plosmedicine.org/, Annals of Internal Medicine at http://www.annals.org/, and Epidemiology at http://www.epidem.com/). Information on the STROBE Initiative is available at www.strobe-statement.org.

For peer review only

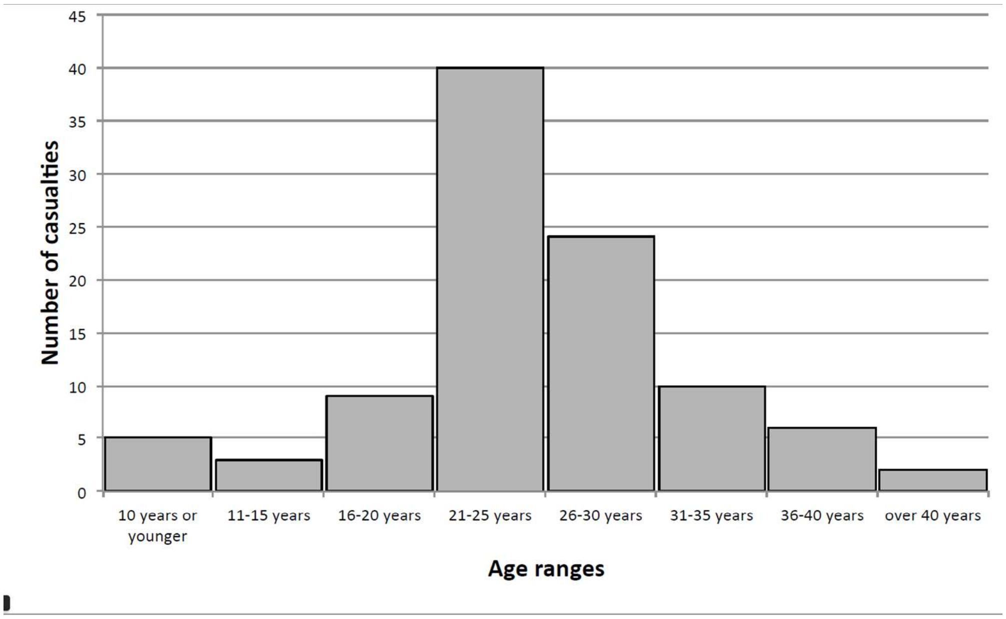

Figure 1: Age ranges of AP-IED targeted victims

125x76mm (300 x 300 DPI)

For peer review only

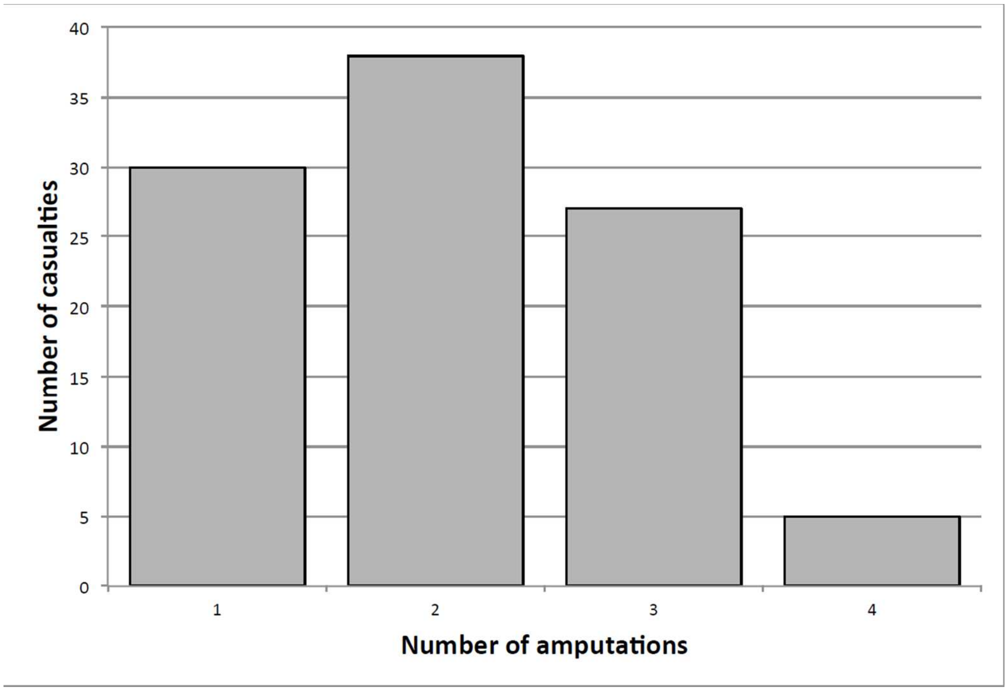

Figure 2: Numbers of traumatic limb amputations per targeted victim of the AP-IED

112x76mm (300 x 300 DPI)

view only

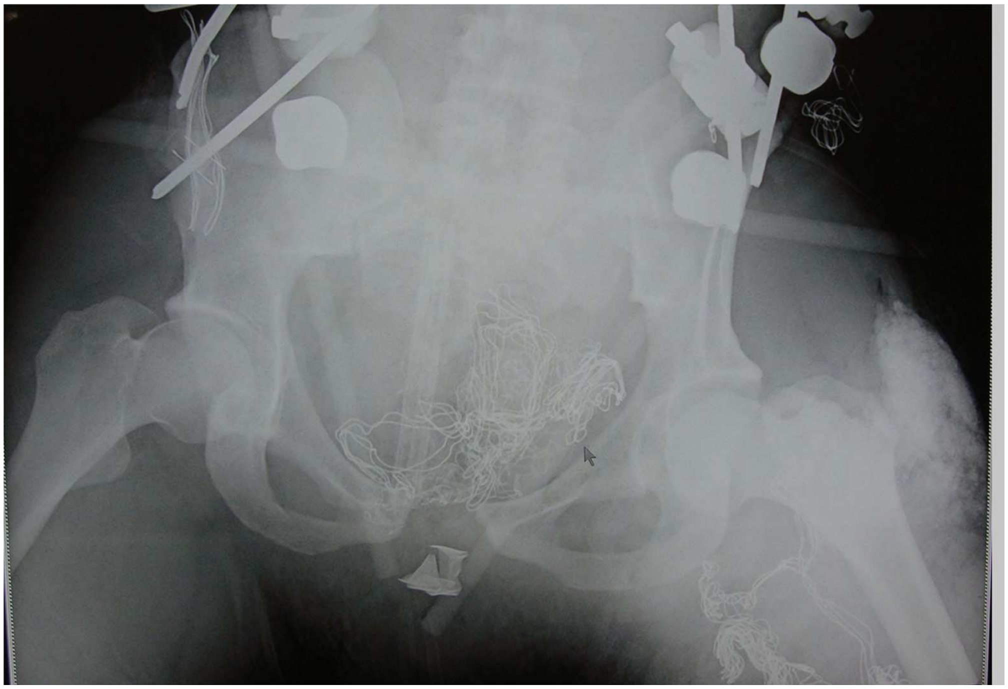

Figure 3: X-ray taken for placement of a pelvic external fixator showing: disruption of pelvic ring by the force of an anti-personnel improvised explosive device; the arrow indicates combat gauze packing a severe perineal injury; silica laden soil injection by the explosion.

440x300mm (300 x 300 DPI)

view only

BMJ Open

Profile of injuries suffered by the target of the antipersonnel improvised explosive device

Journal:	*BMJ Open*
Manuscript ID	bmjopen-2016-014697.R1
Article Type:	Research
Date Submitted by the Author:	14-Feb-2017
Complete List of Authors:	Smith, Shane; Royal Canadian Medical Service; Western University, Surgery<br>Devine, Melissa ; Royal Canadian Medical Service, Critical Care Nursing<br>Taddeo, Joseph; Maine Veterans' Affairs Medical Center, Surgery<br>McAlister, Vivian; Royal Canadian Medical Service, ; Western University, Surgery
**Primary Subject Heading**:	Global health
Secondary Subject Heading:	Emergency medicine, Surgery
Keywords:	blast injury, war, amputation, soft tissue injury, landmines

SCHOLARONE™ Manuscripts

For peer review only

Profile of injuries suffered by the target of the antipersonnel improvised explosive device

Shane Smith[1,2]

Melissa Devine[2]

Joseph Taddeo[3]

Vivian McAlister[1,2*]

[1]Royal Canadian Medical Service

[2]Division of General Surgery, University of Western Ontario, Ontario, Canada

[3]Maine Veterans' Affairs Medical Center, Augusta, Maryland, USA

Shane Smith

Medical Officer, Royal Canadian Medical Service

C4-211 University Hospital, London, Ontario, Canada, N5X 2S1

Melissa Devine

Critical Care Nursing Officer, Royal Canadian Medical Service

QEII Health Science Centre, 1276 South Park Street 3rd Floor Bethune, Room 371

Halifax, NS B3H 2Y9

Joseph Taddeo

General-Trauma Surgeon, Maine Veterans' Affairs Medical Center

1 VA Center Augusta, ME 04330, USA

For peer review only - http://bmjopen.bmj.com/site/about/guidelines.xhtml

For peer review only

Vivian McAlister

General Surgeon, Royal Canadian Medial Service

C8-005 University Hospital, London, Ontario, Canada, N5X 2S1.

Telephone: (519) 663-2920, Fax: 1-(855)-538-2926

Email: *vmcalist@uwo.ca*

***Corresponding author**

Word count: 2,809

Keywords: Improvised Explosive Device; IED; Blast; Amputation; Trauma; Military;

For peer review only

For peer review only - http://bmjopen.bmj.com/site/about/guidelines.xhtml

The views expressed in this paper are those of the authors and do not constitute the views or policies of the Canadian Armed Forces or the United States Navy.

Competing interests: All authors have completed the ICMJE uniform disclosure form at www.icmje.org/coi_disclosure.pdf and declare: no support from any organisation for the submitted work; no financial relationships with any organisations that might have an interest in the submitted work in the previous three years; no other relationships or activities that could appear to have influenced the submitted work.

Contributions: S.S. analysed the data and drafted the manuscript. M.D. conducted data collection. V.M. and J.T. were the treating trauma surgeons of the casualties and designers of the study. They also conducted data collection. The manuscript has been reviewed and approved by all authors. All authors had full access to all of the data in the study and can take responsibility for the integrity of the data and the accuracy of the data analysis.

This study did not receive any outside funding. The Canadian Armed Forces employed S.S., M.D., and V.M. at the time of the study. The United States Navy employed J.D. at the time of the study.

Transparency declaration: The lead author affirms that the manuscript is an honest, accurate, and transparent account of the study being reported; that no important aspects of the study have been omitted; and that any discrepancies from the study as planned have been explained.

For peer review only - http://bmjopen.bmj.com/site/about/guidelines.xhtml

For peer review only

Data sharing: The contact author is prepared to answer queries regarding the trial protocol; statistical code used for comparisons and the data set.

For peer review only - http://bmjopen.bmj.com/site/about/guidelines.xhtml

Objective: To describe Pattern 1 injures caused by the antipersonnel improvised explosive device (AP-IED) in comparison to those previously for anti-personnel mines (APM)

Design: Prospective cohort study of 100 consecutive pedestrian victims of an AP-IED, with a traumatic amputation without regard for gender, nationality or military status.

Setting: Multinational Medical Unit at Kandahar Air Field, Afghanistan.

Participants: One hundred consecutive patients, all male, 6 to 44 years old.

Main outcome measures: The details of injuries including: the level of amputation, the level of soft tissue injury, the fracture pattern (including pelvic fractures) as well as perineal, gluteal, genital, and other injuries were recorded.

Results: Victims of AP-IED were more likely, compared to APM victims, to have multiple amputations (70.0% vs. 10.4%; $p<0.001$) or genital injury (26% vs. 13%; $p=0.007$). Multiple amputations occurred in 70 patients: 5 quadruple, 27 triple, and 38 double amputations. Pelvic fracture occurred in 21 victims, all but one of whom had multiple amputations. Severe perineal, gluteal, or genital injuries were present in 46 patients. Severe soft tissue injury was universal, with injection of contaminated soil along tissue planes well above entry sites. There were 13 facial injuries, 9 skull fractures, and 3 traumatic brain injuries. Eleven eye injuries were seen; none of the victims with eye injuries were wearing eye protection. The casualty fatality rate was at least 19%. The presence of more than one amputation was associated with a higher rate of pelvic fracture (28.6% vs. 3.3%; $p=0.005$) and perineal - gluteal injury (32.6% vs. 11.1%; $p=0.009$).

Conclusion: The injury pattern suffered by the target of the AP-IED is markedly worse than that of conventional APM, resulting in superfluous injury and unnecessary suffering.

For peer review only - http://bmjopen.bmj.com/site/about/guidelines.xhtml

Pelvic binders should be applied at the point of injury to patients with multiple amputations or perineal injuries.

For peer review only

For peer review only - http://bmjopen.bmj.com/site/about/guidelines.xhtml

ARTICLE SUMMARY

Strengths and limitations of this study:

This is a prospective consecutive series with a predefined definition of the target of the AP-IED attack used as inclusion criteria.

The definition of the target of the AP-IED was chosen so as to facilitate comparison to the International Committee of the Red Cross land mine data. This excluded injured bystanders who may have more minor injuries.

The Role 3 Multinational Medical Unit was the only surgical facility in the region. This maximized catchment of AP-IED victims and minimized selection bias.

Patients who died at the scene of the explosion may not have been evacuated to the Role 3 Multinational Medical Unit and victims who died after discharge would not have been included in this study. This may have resulted in an underestimation of the AP-IED's lethality. However studies of APM victims, to which this study is compared, are subject to a similar risk of underestimation.

For peer review only - http://bmjopen.bmj.com/site/about/guidelines.xhtml
For peer review only

INTRODUCTION

Antipersonnel mines (APM) came into widespread use in the Second World War. They were designed to injure but not kill, in order to remove a target from combat and to increase the logistical burden of caring for the casualty. These weapons are typically buried and left for the target to trigger. The weapon is indiscriminate because non-combatants such as children and civilians may detonate them. They are also frequently left buried and active after fighting in that region, or the conflict itself, has ended. Following a public relations campaign that highlighted the indiscriminate injuries caused by APMs, 162 countries signed the 1999 Ottawa treaty ceasing their production and use.[1] An important basis of that campaign was the clinical description of APM related injuries by Robin Coupland and Adriaan Korver for the International Committee of the Red Cross (ICRC).[2] Three patterns of injury were found among 754 victims treated at two ICRC hospitals. In pattern 1 injuries, the victim often triggers the mine by stepping on it and suffers the full effect of the explosion. In pattern 2 injuries, victims are farther away from the centre of the explosion and suffer wounds from fragments. Pattern 3 injuries are caused by handling the mine, resulting in severe injuries to the hands and face. Pattern 1 victims usually suffer one or more traumatic amputations, usually of the lower limb, whereas pattern 2 victims have fragment wounds scattered over the body. Pattern 2 victims may have fragment injuries of the lower limbs but usually the damage is not severe enough to cause traumatic amputation.

This distinguishing feature of a traumatic amputation between pattern 1 and pattern 2 injuries was confirmed in a later report of 4616 APM victims which present further

For peer review only - http://bmjopen.bmj.com/site/about/guidelines.xhtml

evidence of the severity of injury particularly to the limbs.[3] Of 1077 (23%) pattern 1 victims, 606 (56%) had below knee amputation (BKA), 26 (2%) bilateral BKA, 349 (32%) unilateral above the knee amputations (AKA), 57 (5%) bilateral AKA, and 39 (4%) a combination of AKA and BKA.[3] Their description of the typical pattern 1 injury profile caused by an APM, which has been confirmed by other studies, is of a traumatic amputation of the foot or leg with scattered penetrating injuries elsewhere. [2-5]

The improvised explosive device (IED) is increasingly used in modern conflicts including Afghanistan.[6,7] The majority of these weapons have been directed against pedestrian individuals in a similar fashion to APM.[8,9] As with APM, injuries suffered by victims of the anti-personnel IED (AP-IED) depend on whether they were the target of the explosive device or at some distance from the centre of the explosion. It does not depend on the manufacture of the explosive device, industrial as in APM versus improvised as in AP-IED. Indeed elements of conventional explosives may be used to construct an AP-IED. The blast injury depends on the energy transferred by the explosion to the victim. However it was our impression that pattern 1 injuries caused by AP-IED were significantly worse than those reported for APM.[2] The purpose of this paper is to describe the profile of pattern 1 injury suffered by the target of the AP-IED compared to the target of APM.

For peer review only - http://bmjopen.bmj.com/site/about/guidelines.xhtml

METHODS

The target of an AP-IED was defined as the pedestrian victim of an IED with a traumatic amputation. In order to be included in the study, casualties were required to have been reported by the first responder as being the dismounted (pedestrian) victim of an IED explosion and to have suffered a traumatic amputation. Casualties that were non-pedestrian (i.e. in a vehicle) or whose situation was unknown were excluded. The study was designed to describe the injuries suffered by 100 consecutive targets (pattern 1) of AP-IED attacks who presented to the North Atlantic Treaty Organization Role 3 Multinational Medical Unit (R3-MMU) in Kandahar Air Field (KAF). The hospital received casualties from the point of injury or via a forward treatment centre.[10] A R3-MMU is the highest level of in-theatre medical care and would be equivalent to a civilian level-II trauma centre, as defined by the American College of Surgeons-Committee On Trauma. A specifically designated trauma nurse (M.D.), and attending surgeons (J.T., V.M.) prospectively collected data for each patient. Data was collected between January 2010 and July 2011, or until data from 100 victims had been obtained. Data collected included: nationality, age, gender, mortality status, specific injuries, and imaging results. The level of traumatic amputation, the level of soft tissue injury, the fracture pattern (including pelvic fractures) as well as perineal and gluteal injuries were specifically recorded. Generally, coalition patients were transferred after damage control surgery, whereas local patients also received definitive care at the R3-MMU. Data were collected until the time of first discharge from the hospital. In order to minimize bias, patients were included sequentially as a consecutive series with a predefined definition of AP-IED target. The R3-MMU was the only surgical facility in the region and would receive all

AP-IED victims for care. Patient level data is reported identifying the injuries of each of the 100 victims. The paediatric victims are also presented separately. The data collection was complete with injury data captured for all 100 victims. Categorical data was analysed using the chi-square test. AP-IED data was compared to previously published APM data[2].This study was approved by the commander of the R3-MMU KAF and by the Research Ethics Board of the University of Western Ontario (REB # 104124).

RESULTS

One hundred consecutive casualties with amputations from AP-IEDs were identified and their injuries were described. All of the patients were male. The mean, and median, age of the victims was 25 years. The age range was from ages 6 to 44 years. There were 9 patients under the age of 18 (Figure 1). Twenty-seven patients were Afghan local nationals and 61 were coalition soldiers (USA, Canada, UK); there were 12 victims whose nationality was not captured; approximately 17% of the patients were designated as civilians (Table 1). Eleven patients were dead upon arrival and another 8 died of their wounds in the hospital, giving a 19% casualty fatality rate. Gender, injury pattern, age or approximate age, was recorded for all victims.

AP-IED victims were more likely than a similar cohort of APM victims to suffer multiple amputations (70.0% vs. 10.4%; $p<0.001$) or genital injury (26.0% vs. 13.4%; $p=0.007$). Seventy victims (70%) sustained multiple amputations: 5 patients suffered quadruple amputations, 27 had triple amputations, and 38 victims had double amputations (Figure 2). Two victims had hip disarticulations, 41 had at least one AKA, and 56 had at least one

For peer review only - http://bmjopen.bmj.com/site/about/guidelines.xhtml

BKA (including through knee and through ankle amputations). Sixty-five victims had bilateral lower extremity amputations and 37 had at least one upper extremity amputation (at the level of the hand or higher) (Table 2). Pelvic fractures were present in 21 victims, all but one of whom had multiple amputations. Forty-six patients had perineal and gluteal injuries; these included 8 anorectal injuries, 8 penile injuries, and 26 scrotal injuries including 10 traumatic orchiectomies. There were 13 facial injuries, 9 skull fractures and 3 traumatic brain injuries. Eye injuries were found in 11 patients, none of whom were wearing eye protection. Of the 9 pediatric patients, 3 had triple amputations and 5 had double amputations. There was one pelvic fracture and two perineal injuries among the pediatric victims (Table 3). Pelvic fracture was more likely if the victim had multiple amputations (28.6% vs. 3.3%; p=0.005) or had a perineal or gluteal injury (32.6% vs. 11.1%; p=0.009). Those victims with multiple amputations also had more perineal and gluteal injuries (52.9% vs. 30%; p=0.036) and a higher mortality (24.3% vs. 6.7%; p=0.039).

Therefore, the typical injury profile suffered by targeted victims of AP-IEDs included: bilateral lower extremity amputations (often above the knee); mangling or amputation of an upper extremity; extensive soft tissue injury with deep contamination by soil, extending into gluteal and perineal regions; pelvic ring disruption and genital mutilation.

DISCUSSION

The mechanism of injury is the same for all anti-personnel explosive devices. The severity of injury depends upon the energy transferred by the explosion to the victim.

For peer review only - http://bmjopen.bmj.com/site/about/guidelines.xhtml

There is nothing inherently different between the APM and the AP-IED. Indeed the AP-IED might be considered a subset of APM. However the comparison in this study is between the conventional APM, manufactured industrially for national armies, and the AP-IED, manufactured locally from available materials by irregular forces. Coupland and Korver believed they were dealing with the former device.[2,3,4] In a textbook reflecting on the Russian experience in Afghanistan, Bruysov and colleagues illustrated the mechanism of injury from the conventional APM in a series of experiments using rapid sequence photography which confirmed Coupland and Korver's description of a foot amputation with scattered penetrating injuries elsewhere.[11]

The AP-IED, sometimes portrayed as a primitive or crude weapon crafted from locally available resources because of a lack of access to conventional weapons, has evolved with use in different conflicts. From rudimentary devices of nails in wooden boxes used in Cambodia and Columbia, AP-IEDs used in recent conflicts are better directed and more destructive. The injury profile from the modern AP-IED is far worse than originally described for conventional APMs. Whereas the APM typically results in a unilateral lower limb amputation, the modern AP-IED causes bilateral high amputation of the lower extremities. Triple amputations were not seen with APM but occurred in 27% of victims of AP-IEDs. Severe perineal and gluteal injury with soft tissue contamination with soil was commonly present in the victims of the AP-IED but is not seen among APM injuries. The energy transfer endured by targeted victims of AP-IEDs must be far greater than that caused by APMs. A measure of the force is the fact that it is sufficiently powerful to disrupt the pelvic ring in one fifth of the patients.

For peer review only - http://bmjopen.bmj.com/site/about/guidelines.xhtml

It is not the purpose of this paper to undermine the abhorrence of injuries caused by conventional APMs, which are often devastating and ruin lives. Both the APM and AP-IED injure indiscriminately. In this series, almost one in ten victims of AP-IEDs was a child, a tragic feature shared with APMs. Even more disconcerting is the frequency with which children suffer the severest injuries from the powerful explosive force of the AP-IED: 89% suffered multiple amputations and 33% lost three limbs.

The AP-IED victims wore different types of personal protective equipment (PPE) or none at all. Coalition soldiers wore PPE including helmets, body armour, and anti-ballistic eyewear. Each nation issued its own style and brand of PPE. Local Afghan soldiers often used helmets but their use of body armour was more inconsistent and almost never included anti-ballistic eyewear. Civilian casualties were not wearing any PPE. This heterogeneity of PPE use in our sample may have affected the pattern of injury characterized in this study but the sample size was insufficient to determine accurately the injury preventative role of PPE. We felt there was a lower than expected rate of abdominal, thoracic and eye injuries in victims wearing PPE. We also had the impression that the design purpose of PPE, to prevent fragment injury, was confirmed in our experience of patients with pattern 2 injuries but they are not included in this study.

It is possible that we have magnified the severity of the injury pattern by concentrating our focus upon patients that met our definition of being targeted by the AP-IED, that is having sustained at least one limb amputation. It is also possible that if a casualty was injured by an unexploded ordinance or classical land mine but reported by first responders to have been injured by an AP-IED they may have been erroneously included.

However our findings are in keeping with other descriptions of IED injuries in the literature. Jacobs *et al*. classified the limb injury pattern of 103 consecutive casualties of IEDs treated at a UK Role 3 facility in Helmand Province, Afghanistan.[12] They included victims who did not have amputation of a limb. They found 76 victims suffered significant bilateral lower limb injuries, with 50 who required bilateral lower limb amputation. Thirty-three victims suffered genital or perineal injury, 9 sustained pelvic ring disruption, and 40 sustained significant upper limb injury. They found that all pelvic fractures and 80% of genital injuries were sustained among casualties with bilateral lower limb amputations. A retrospective review of the UK Joint Theatre Trauma Registry examined UK services personnel who were casualties of IEDs in Afghanistan, who sustained lower extremity amputations between January 2007 and December 2010.[13] This registry includes post-mortem reports of soldiers who were killed and never received hospital care. The registry is restricted to UK services members and contains no local nationals or civilian injuries. They examined 656 IED victims with 138 killed in action (21%) and 31 who died of their wounds (4.7%) resulting in a 25.9% casualty fatality rate. Of the 169 victims who sustained a traumatic lower extremity amputation, 69 were killed in action (40.8%) and 31 died of their wounds (18.3%). They found that the level of amputation was inversely correlated with survival with only two survivors from hindquarter amputation out of 39 patients with this level of injury. Another retrospective review of the UK Military Trauma Registry focused on soldiers from Afghanistan with bilateral leg amputation.[8] They examined 43 casualties with at least two amputations; 80% of these were from AP-IEDs with the other 20% being from anti-vehicle-IEDs. The most common bilateral amputation was a bilateral AKA (58%), and 20% of victims

For peer review only - http://bmjopen.bmj.com/site/about/guidelines.xhtml

suffered triple amputation. 14% of victims sustained open pelvic fractures and 44% suffered perineal or genital injury. No victims survived the loss of all four limbs.

The soft tissue injury is a particularly difficult problem encountered by those caring for victims of AP-IEDs. The directed explosion forces soil up along soft tissue planes far above the point of entry. This may worsen the level of eventual amputation and it condemns the victim to multiple operations to remove the contamination. Even still, it may leave the victim at the mercy of unusual antibiotic resistant soil organisms such as *Acinetobacter baumannii.*[14]

Knowing the pattern of injury helps responders tailor care. As Jacobs and colleagues have suggested, first responders should apply tourniquets bilaterally even if the victim is not bleeding, as haemorrhage is likely to start once resuscitation restores intravascular volume.[12] While current protocols require medics to apply a neck collar to all victims of IEDs, spinal injury is rare in AP-IED victims despite the magnitude of the force.[9] On the other hand, we recommend that all AP-IED victims with perineal injuries or multiple amputations have pelvic binders placed by the first responders, as both are associated with pelvic fracture. This may reduce the large amount of blood that may be lost silently with pelvic disruption. Multiple surgical teams have to work simultaneously to rescue these patients. Surgeons have to be adept at dealing with pelvic and gluteal bleeding, sometimes by pre-peritoneal packing and by ligating the internal iliac artery. Rehabilitation requires mobilization on temporary lower limb prostheses despite injured upper limbs. Finally, victims of this severe injury pattern need life-long support because

For peer review only - http://bmjopen.bmj.com/site/about/guidelines.xhtml

disabilities may become more pronounced as the capacity to compensate fades with age.

In collecting information on 100 consecutive patients, we hope to have described a generalizable result. Due to the nature of the conflict and the local culture, all of the patients examined were male. While we would expect the severity of the injury pattern to be generalizable to females, we cannot explicitly determine the effect of the modern AP-IED on the female pelvis and perineum.

The use of conventional APM has decreased in the last 20 years since the campaign to prohibit their use. Pattern 1 injuries seen in recent conflicts are far more often from AP-IEDs than conventional APMs. ICRC includes AP-IEDs in its definition of APM. The injury profile described in this report is illustrated in the recent edition of its textbook on war surgery.[15] Despite the huge force endured by the targeted victim of the AP-IED, the casualty fatality rate that we observed was 19%. This rate is an underestimate, as some casualties who were killed at the site of the AP-IED may not have been brought to our facility and others may have died as a result of injuries after transfer or discharge. Reports regarding APM are also affected by this problem. The observed mortality rate with AP-IED is of the same order or less than that elsewhere.[15] This information has a disturbing implication. The military purpose of the AP-IED has not been studied. It would appear to be similar to the conventional APM: to disable but not kill an opponent. However the modern AP-IED, by its nature, causes superfluous injury and unnecessary suffering. Just as the reports by Coupland and Korver provided the medical evidence upon which the prohibition of conventional APM was based, it is hoped that reports regarding the pattern of injury caused by the modern AP-IED will result in an abhorrence of this weapon and

For peer review only - http://bmjopen.bmj.com/site/about/guidelines.xhtml

those who use it.

For peer review only - http://bmjopen.bmj.com/site/about/guidelines.xhtml

REFERENCES

1. United Nations. Convention on the Prohibition of the Use, Stockpiling, Production and Transfer of Anti-Personnel Mines and on their Destruction. *United Nations, Treaty Series , vol 2056, p 211.* 1999.

2. Coupland RM, Korver A. Injuries from antipersonnel mines: the experience of the International Committee of the Red Cross. *BMJ* 1991;303(6816):1509-1512.

3. Korver AJ. Injuries of the lower limbs caused by antipersonnel mines: the experience of the International Committee of the Red Cross. *Injury.* 1996;27(7):477-479.

4. Coupland RM. Amputation for antipersonnel mine injuries of the leg: preservation of the tibial stump using a medial gastrocnemius myoplasty. *Annals of the Royal College of Surgeons of England.* 1989;71(6):405-408.

5. Soroush A, Falahati F, Zargar M, Soroush M, Khateri S, Khaji A. Amputations due to landmine and unexploded ordinances in post-war Iran. *Archives of Iranian Medicine.* 2008;11(6):595-597.

6. Jacobs N, Taylor DM, Parker PJ. Changes in surgical workload at the JF Med Gp Role 3 Hospital, Camp Bastion, Afghanistan, November 2008-November 2010. *Injury.* 2012;43(7):1037-1040.

7. Beckett A, Pelletier P, Mamczak C, Benfield R, Elster E. Multidisciplinary trauma team care in Kandahar, Afghanistan: current injury patterns and care practices. *Injury.* 2012;43(12):2072-2077.

For peer review only - http://bmjopen.bmj.com/site/about/guidelines.xhtml

8. Penn-Barwell JG, Bennett PM, Kay A, Sargeant ID. Acute bilateral leg amputation following combat injury in UK servicemen. *Injury.* 2014;45(7):1105-1110.

9. Taddeo J, Devine M, McAlister VC. Cervical spine injury in dismounted improvised explosive device trauma. *Can J Surg.* 2015;58(3 Suppl 3):S104-107.

10. Brisebois R, Hennecke P, Kao R, et al. The Role 3 Multinational Medical Unit at Kandahar Airfield 2005-2010. *Can J Surg.* 2011;54(6):S124-129.

11. Bryusov PG, Shapovalov VM, Artemyev AA, Dulayev AK, Gololobov VG. Combat Injuries of Extremities. Moscow: Military Medical Academy, GEOTAR; 1996. [Translation by ICRC Delegation, Moscow]

12. Jacobs N, Rourke K, Rutherford J, et al. Lower limb injuries caused by improvised explosive devices: proposed 'Bastion classification' and prospective validation. *Injury.* 2014;45(9):1422-1428.

13. Morrison JJ, Hunt N, Midwinter M, Jansen J. Associated injuries in casualties with traumatic lower extremity amputations caused by improvised explosive devices. *The British Journal of Surgery.* 2012;99(3):362-366.

14. O'Shea MK. Acinetobacter in modern warfare. *Int J Antimicrob Agents.* 2012;39(5):363-375.

15. Giannou C, Baldan M, Molde A. War Surgery: working with limited resources in armed conflict and other situations of violence (Volume 2). Geneva: International Committee of the Red Cross; 2103. <https://www.icrc.org/eng/assets/files/publications/icrc-002-4105.pdf>

For peer review only - http://bmjopen.bmj.com/site/about/guidelines.xhtml

Table 1: Victim characteristics

Characteristic	
Number of patients	100
Age, mean (SD)	25 (6.8) yrs
Median	25 yrs
Range	6 – 44 yrs
Male	100
Female	0
Nationality	
Afghan	27
Coalition	61
American	49
Canadian	8
British	4
Not specified	12
Mortality	19
Killed in Action	11
Died of wounds	8

SD = standard deviation

For peer review only - http://bmjopen.bmj.com/site/about/guidelines.xhtml

Table 2: Distribution of amputations

Amputation Type	Overall	Pelvic Fracture	Perineal, Gluteal, Genital Injury	Killed in Action	Died of Wounds	Casualty Fatality Rate
HipDis/TKA/UEA	1	0	0	0	1	1/1
HipDis/-	1	0	0	0	1	1/1
AKA/AKA/UEA/UEA	4	0	3	0	0	0/4
AKA/AKA/UEA	7	3	6	0	0	0/7
AKA/AKA	8	2	4	2	1	3/8
AKA/BKA/UEA	2	1	0	1	1	2/2
AKA/TKA/UEA	1	1	0	0	1	1/1
AKA/BKA	7	1	5	0	0	0/7
AKA/TKA	7	4	6	0	0	0/7
AKA/UEA/UEA	1	0	0	0	0	0/1
AKA/UEA	1	0	0	0	0	0/1
AKA/TAA	1	0	0	0	0	0/1
AKA/-	2	0	0	0	0	0/2
BKA/BKA/UEA/UEA	1	0	0	0	1	1/1
BKA/BKA/UEA	13	4	6	4	2	6/13
BKA/TKA/UEA	1	0	1	0	0	0/1
BKA/BKA	9	3	5	2	0	2/9
BKA/UEA	2	0	0	0	0	0/2
BKA/TKA	1	0	0	1	0	1/1
BKA/-	22	1	9	1	0	1/22
TKA/TKA/UEA	1	1	1	0	0	0/1
TKA/TKA	1	0	0	0	0	0/1
TKA/-	1	0	0	0	0	0/1
TAA/UEA	1	0	0	0	0	0/1
TAA/-	3	0	0	0	0	0/3
UEA/-	1	0	0	0	0	0/1

HipDis, hip disarticulation; AKA, above knee amputation; BKA, below knee amputation; TKA, though knee amputation, TAA, though ankle amputation; UEA, upper extremity amputation, which includes above elbow, below elbow and hand amputations.

Table 3: Distribution of pediatric amputations

Age	Amputation Type	Pelvic Fracture	Perineal, Gluteal, Genital Injury	Disposition
16	AKA/BKA	No	No	Survived
14	AKA/AKA	No	Yes	DOW
14	AKA/UEA/UEA	No	No	Survived
11	UEA/TKA/AKA	Yes	No	DOW
10	BKA/BKA/UEA	No	No	KIA
10	UEA/TAA	No	No	Survived
10	BKA/AKA	No	Yes	Survived
10	BKA/BKA	No	No	KIA
6	UEA	No	No	Survived

AKA, above knee amputation; BKA, below knee amputation; TKA, though knee amputation, TAA, though ankle amputation; UEA, upper extremity amputation, which includes above elbow, below elbow and hand amputations; DOW, died of wounds; KIA, killed in action.

For peer review only - http://bmjopen.bmj.com/site/about/guidelines.xhtml

Figure 1: Victim ages

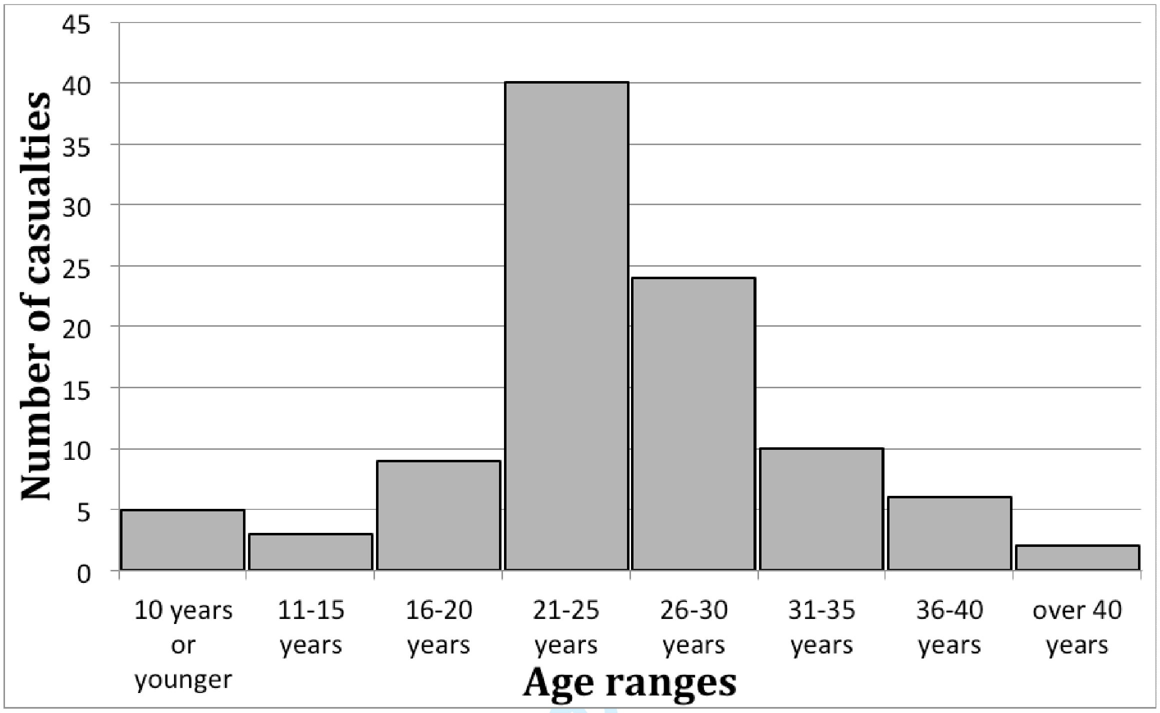

For peer review only

Figure 2: Number of amputations per victim

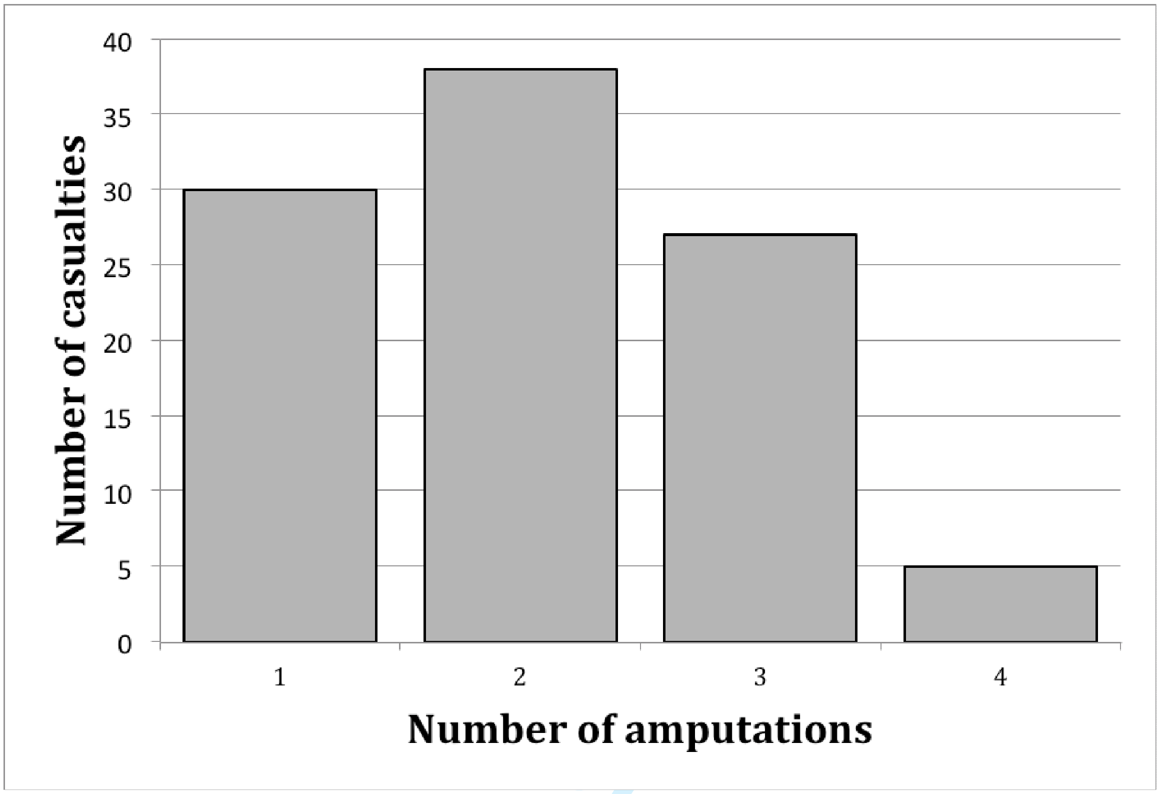

For peer review only - http://bmjopen.bmj.com/site/about/guidelines.xhtml

Figure 3:

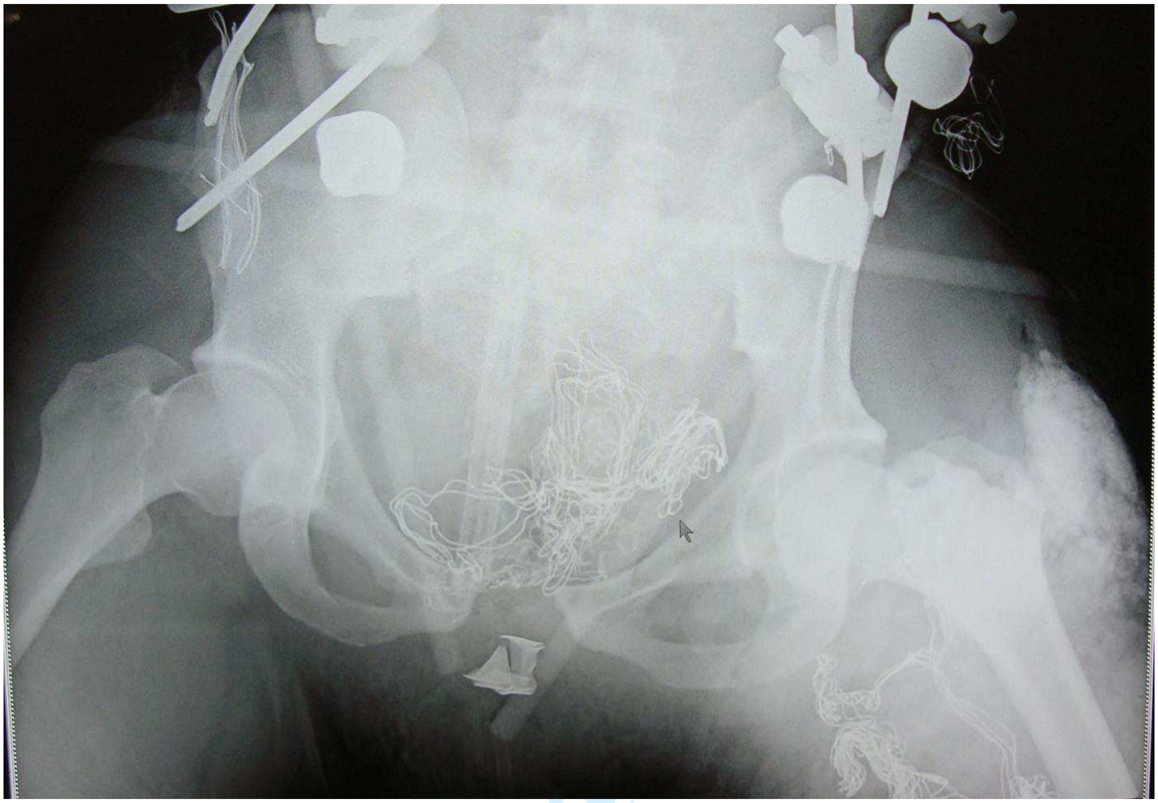

For peer review only - http://bmjopen.bmj.com/site/about/guidelines.xhtml

Legend for figures

Figure 1: Age ranges of AP-IED targeted victims

Figure 2: Numbers of traumatic limb amputations per targeted victim of the AP-IED

Figure 3: X-ray taken for placement of a pelvic external fixator showing: disruption of pelvic ring by the force of an anti-personnel improvised explosive device; the arrow indicates combat gauze packing a severe perineal injury; silica laden soil injection by the explosion.

For peer review only

For peer review only - http://bmjopen.bmj.com/site/about/guidelines.xhtml

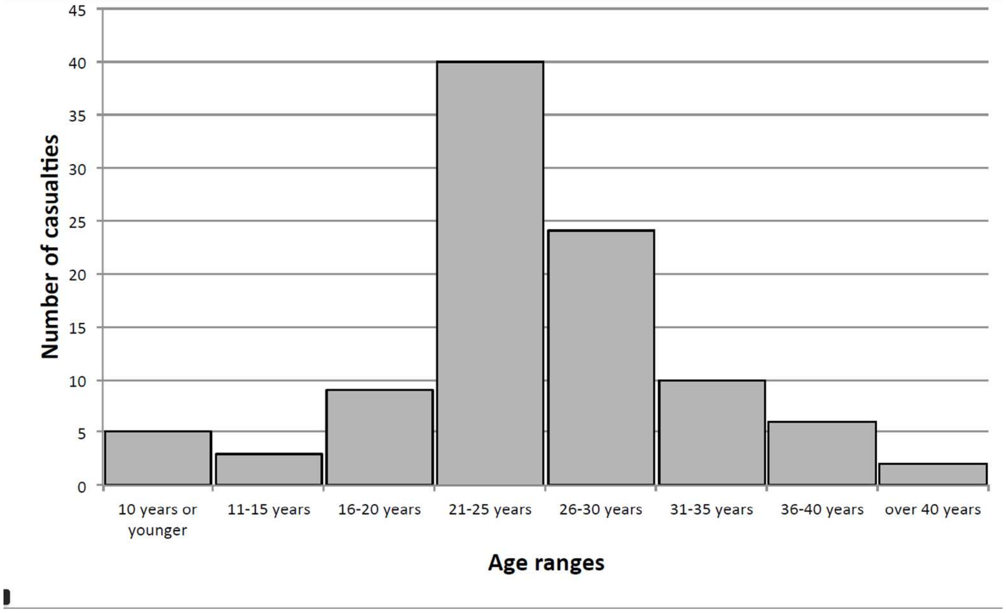

Figure 1: Age ranges of AP-IED targeted victims

125x76mm (300 x 300 DPI)

For peer review only

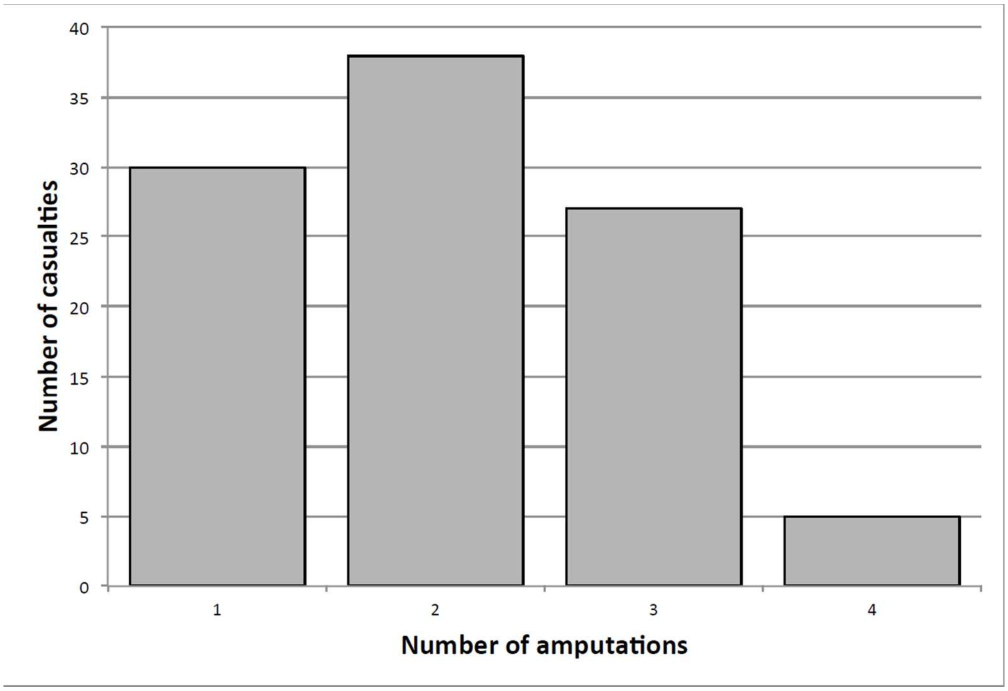

Figure 2: Numbers of traumatic limb amputations per targeted victim of the AP-IED

112x76mm (300 x 300 DPI)

view only

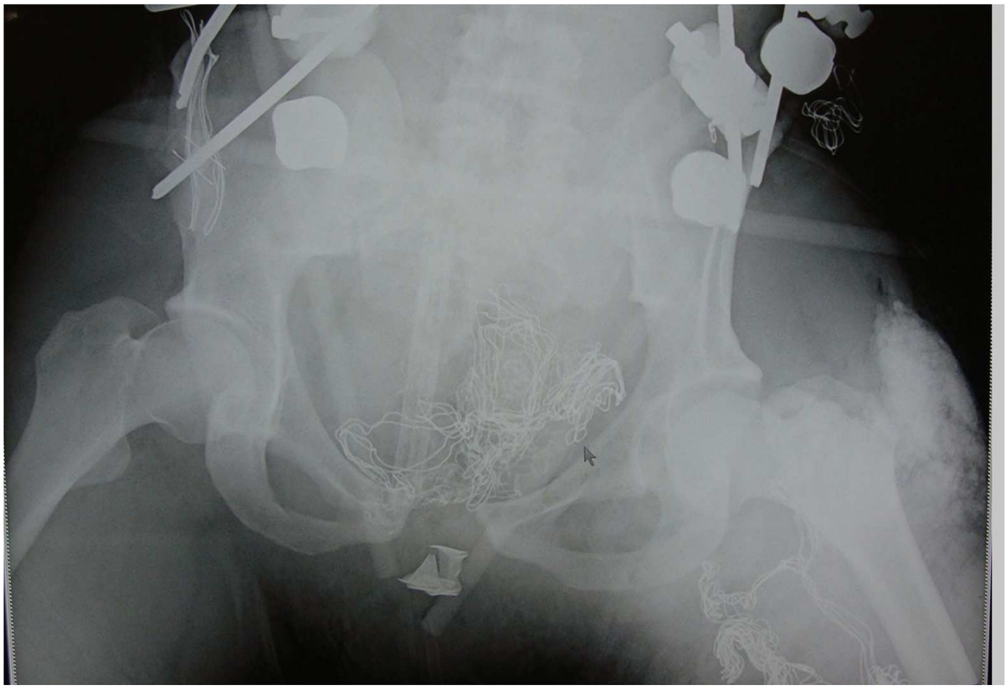

Figure 3: X-ray taken for placement of a pelvic external fixator showing: disruption of pelvic ring by the force of an anti-personnel improvised explosive device; the arrow indicates combat gauze packing a severe perineal injury; silica laden soil injection by the explosion.

440x300mm (300 x 300 DPI)

view only

STROBE Statement—checklist of items that should be included in reports of observational studies

	Item No	Recommendation
**Title and abstract**	1	(*a*) Indicate the study's design with a commonly used term in the title or the abstract **Pg 4**
		(*b*) Provide in the abstract an informative and balanced summary of what was done and what was found **Pg 4**
**Introduction**		
Background/rationale	2	Explain the scientific background and rationale for the investigation being reported **Pg 7-8**
Objectives	3	State specific objectives, including any prespecified hypotheses **Pg 9**
**Methods**		
Study design	4	Present key elements of study design early in the paper **Pg 9**
Setting	5	Describe the setting, locations, and relevant dates, including periods of recruitment, exposure, follow-up, and data collection **Pg 9**
Participants	6	(*a*) *Cohort study*—Give the eligibility criteria, and the sources and methods of selection of participants. Describe methods of follow-up **Pg 9**<br>*Case-control study*—Give the eligibility criteria, and the sources and methods of case ascertainment and control selection. Give the rationale for the choice of cases and controls<br>*Cross-sectional study*—Give the eligibility criteria, and the sources and methods of selection of participants
		(*b*) *Cohort study*—For matched studies, give matching criteria and number of exposed and unexposed N/A<br>*Case-control study*—For matched studies, give matching criteria and the number of controls per case
Variables	7	Clearly define all outcomes, exposures, predictors, potential confounders, and effect modifiers. Give diagnostic criteria, if applicable **Pg 9**
Data sources/ measurement	8*	For each variable of interest, give sources of data and details of methods of assessment (measurement). Describe comparability of assessment methods if there is more than one group **Pg 9**
Bias	9	Describe any efforts to address potential sources of bias **Pg 10**
Study size	10	Explain how the study size was arrived at **Pg 9**
Quantitative variables	11	Explain how quantitative variables were handled in the analyses. If applicable, describe which groupings were chosen and why **N/A**
Statistical methods	12	(*a*) Describe all statistical methods, including those used to control for confounding **Pg 10**
		(*b*) Describe any methods used to examine subgroups and interactions **Pg 10**
		(*c*) Explain how missing data were addressed **Pg 10**
		(*d*) *Cohort study*—If applicable, explain how loss to follow-up was addressed<br>*Case-control study*—If applicable, explain how matching of cases and controls was addressed<br>*Cross-sectional study*—If applicable, describe analytical methods taking account of sampling strategy
		(*e*) Describe any sensitivity analyses

Continued on next page

For peer review only

For peer review only - http://bmjopen.bmj.com/site/about/guidelines.xhtml

**Results**		
Participants	13*	(a) Report numbers of individuals at each stage of study—eg numbers potentially eligible, examined for eligibility, confirmed eligible, included in the study, completing follow-up, and analysed **Pg 10**
		(b) Give reasons for non-participation at each stage
		(c) Consider use of a flow diagram
Descriptive data	14*	(a) Give characteristics of study participants (eg demographic, clinical, social) and information on exposures and potential confounders **Pg 10**
		(b) Indicate number of participants with missing data for each variable of interest **Pg 10 – Table 1**
		(c) *Cohort study*—Summarise follow-up time (eg, average and total amount)
Outcome data	15*	*Cohort study*—Report numbers of outcome events or summary measures over time
		Case-control study—Report numbers in each exposure category, or summary measures of exposure
		Cross-sectional study—Report numbers of outcome events or summary measures
Main results	16	(*a*) Give unadjusted estimates and, if applicable, confounder-adjusted estimates and their precision (eg, 95% confidence interval). Make clear which confounders were adjusted for and why they were included **Pg 10-11**
		(*b*) Report category boundaries when continuous variables were categorized
		(*c*) If relevant, consider translating estimates of relative risk into absolute risk for a meaningful time period
Other analyses	17	Report other analyses done—eg analyses of subgroups and interactions, and sensitivity analyses
**Discussion**		
Key results	18	Summarise key results with reference to study objectives **Pg 12**
Limitations	19	Discuss limitations of the study, taking into account sources of potential bias or imprecision. Discuss both direction and magnitude of any potential bias **Pg 12**
Interpretation	20	Give a cautious overall interpretation of results considering objectives, limitations, multiplicity of analyses, results from similar studies, and other relevant evidence **Pg 13-14**
Generalisability	21	Discuss the generalisability (external validity) of the study results **Pg 15**
**Other information**		
Funding	22	Give the source of funding and the role of the funders for the present study and, if applicable, for the original study on which the present article is based **Pg 3**

*Give information separately for cases and controls in case-control studies and, if applicable, for exposed and unexposed groups in cohort and cross-sectional studies.

Note: An Explanation and Elaboration article discusses each checklist item and gives methodological background and published examples of transparent reporting. The STROBE checklist is best used in conjunction with this article (freely available on the Web sites of PLoS Medicine at http://www.plosmedicine.org/, Annals of Internal Medicine at http://www.annals.org/, and Epidemiology at http://www.epidem.com/). Information on the STROBE Initiative is available at www.strobe-statement.org.

For peer review only

BMJ Open

Injury profile suffered by targets of the antipersonnel improvised explosive device

Journal:	*BMJ Open*
Manuscript ID	bmjopen-2016-014697.R2
Article Type:	Research
Date Submitted by the Author:	24-May-2017
Complete List of Authors:	Smith, Shane; Royal Canadian Medical Service; Western University, Surgery<br>Devine, Melissa ; Royal Canadian Medical Service, Critical Care Nursing<br>Taddeo, Joseph; Maine Veterans' Affairs Medical Center, Surgery<br>McAlister, Vivian; Royal Canadian Medical Service, ; Western University, Surgery
**Primary Subject Heading**:	Global health
Secondary Subject Heading:	Emergency medicine, Surgery
Keywords:	blast injury, war, amputation, soft tissue injury, landmines

SCHOLARONE™
Manuscripts

Injury profile suffered by targets of the antipersonnel improvised explosive device

Shane Smith[1,2]

Melissa Devine[2]

Joseph Taddeo[3]

Vivian McAlister[1,2*]

[1]Royal Canadian Medical Service

[2]Division of General Surgery, University of Western Ontario, Ontario, Canada

[3]Maine Veterans' Affairs Medical Center, Augusta, Maryland, USA

Shane Smith

Medical Officer, Royal Canadian Medical Service

C4-211 University Hospital, London, Ontario, Canada, N6A 5A5

Melissa Devine

Critical Care Nursing Officer, Royal Canadian Medical Service

QEII Health Science Centre, 1276 South Park Street 3rd Floor Bethune, Room 371

Halifax, NS B3H 2Y9

Joseph Taddeo

General-Trauma Surgeon, Maine Veterans' Affairs Medical Center

1 VA Center Augusta, ME 04330, USA

For peer review only - http://bmjopen.bmj.com/site/about/guidelines.xhtml

Vivian McAlister

General Surgeon, Royal Canadian Medial Service

C8-005 University Hospital, London, Ontario, Canada, N6A 5A5

Telephone: (519) 663-2920, Fax: 1-(855)-538-2926

Email: *vmcalist@uwo.ca*

***Corresponding author**

Word count: 2,809

Keywords: Improvised Explosive Device; IED; Blast; Amputation; Trauma; Military;

For peer review only

The views expressed in this paper are those of the authors and do not constitute the views or policies of the Canadian Armed Forces or the United States Navy.

Competing interests: All authors have completed the ICMJE uniform disclosure form at www.icmje.org/coi_disclosure.pdf and declare: no support from any organisation for the submitted work; no financial relationships with any organisations that might have an interest in the submitted work in the previous three years; no other relationships or activities that could appear to have influenced the submitted work.

Contributions: S.S. analysed the data and drafted the manuscript. M.D. conducted data collection. V.M. and J.T. were the treating trauma surgeons of the casualties and designers of the study. They also conducted data collection. The manuscript has been reviewed and approved by all authors. All authors had full access to all of the data in the study and can take responsibility for the integrity of the data and the accuracy of the data analysis.

This study did not receive any outside funding. The Canadian Armed Forces employed S.S., M.D., and V.M. at the time of the study. The United States Navy employed J.D. at the time of the study.

Transparency declaration: The lead author affirms that the manuscript is an honest, accurate, and transparent account of the study being reported; that no important aspects of the study have been omitted; and that any discrepancies from the study as planned have been explained.

For peer review only - http://bmjopen.bmj.com/site/about/guidelines.xhtml

Data sharing: The contact author is prepared to answer queries regarding the trial protocol; statistical code used for comparisons and the data set.

For peer review only

For peer review only - http://bmjopen.bmj.com/site/about/guidelines.xhtml

ABSTRACT

Objective: To describe Pattern 1 injuries caused by the antipersonnel improvised explosive device (AP-IED) in comparison to those previously for anti-personnel mines (APM)

Design: Prospective cohort study of 100 consecutive pedestrian victims of an AP-IED, with traumatic amputation without regard for gender, nationality or military status.

Setting: Multinational Medical Unit at Kandahar Air Field, Afghanistan.

Participants: One hundred consecutive patients, all male, 6 to 44 years old.

Main outcome measures: The details of injuries were recorded in order to describe the pattern and characterize the injuries suffered by the target of AP-IEDs. The level of amputation, the level of soft tissue injury, the fracture pattern (including pelvic fractures) as well as perineal, gluteal, genital, and other injuries were recorded.

Results: Victims of AP-IED were more likely, compared to APM victims, to have multiple amputations (70.0% vs. 10.4%; $p<0.001$) or genital injury (26% vs. 13%; $p=0.007$). Multiple amputations occurred in 70 patients: 5 quadruple amputations, 27 triple amputations, and 38 double amputations. Pelvic fracture occurred in 21 victims, all but one of whom had multiple amputations. Severe perineal, gluteal, or genital injuries were present in 46 patients. Severe soft tissue injury was universal, with injection of contaminated soil along tissue planes well above entry sites. There were 13 facial injuries, 9 skull fractures, and 3 traumatic brain injuries. Eleven eye injuries were seen; none of the victims with eye injuries were wearing eye protection. The casualty fatality rate was at least 19%. The presence of more than one amputation was associated with a

For peer review only - http://bmjopen.bmj.com/site/about/guidelines.xhtml

higher rate of pelvic fracture (28.6% vs. 3.3%; p=0.005) and perineal - gluteal injury (32.6% vs. 11.1%; p=0.009).

Conclusion: The injury pattern suffered by the target of the AP-IED is markedly worse than that of conventional APM. Pelvic binders and tourniquets should be applied at the point of injury to patients with multiple amputations or perineal injuries.

For peer review only

For peer review only - http://bmjopen.bmj.com/site/about/guidelines.xhtml

ARTICLE SUMMARY

Strengths and limitations of this study:

This is a prospective consecutive series with a predefined definition of the target of the AP-IED attack used as inclusion criteria.

The definition of the target of the AP-IED was chosen so as to facilitate comparison to the International Committee of the Red Cross land mine data. This excluded injured bystanders who may have more minor injuries.

The Role 3 Multinational Medical Unit was the only surgical facility in the region. This maximized catchment of AP-IED victims and minimized selection bias.

Patients who died at the scene of the explosion may not have been evacuated to the Role 3 Multinational Medical Unit and victims who died after discharge would not have been included in this study. This may have resulted in an underestimation of the AP-IED's lethality. However studies of APM victims, to which this study is compared, are subject to a similar risk of underestimation.

For peer review only - http://bmjopen.bmj.com/site/about/guidelines.xhtml

INTRODUCTION

Antipersonnel mines (APM) came into widespread use in the Second World War. Originally they were used to protect anti-tank mines, but then went on to become a weapon system in and of themselves. They were designed to injure but not kill, in order to remove a target from combat and to increase the logistical burden of caring for the casualty. These weapons are typically buried and left for the target to trigger. The weapon is indiscriminate because non-combatants such as children and civilians may detonate them. They are also frequently left buried and active after fighting in that region, or the conflict itself, has ended. Following a public relations campaign that highlighted the indiscriminate injuries cause by APMs, 162 countries signed the 1999 Ottawa treaty ceasing their production and use.[1] An important basis of that campaign was the clinical description of APM related injuries by Robin Coupland and Adriaan Korver for the International Committee of the Red Cross (ICRC).[2] Three patterns of injury were found among 754 victims treated at two ICRC hospitals following wounding by blast or fragmentation mines. In pattern 1 injuries, the victim triggers a blast mine by stepping on it and suffers the full effect of the explosion. In pattern 2 injuries, victims are farther away from the centre of the explosion, whether it is a blast or fragmentation mine, and suffer wounds from fragments. Pattern 3 injuries are caused by handling the mine (blast or fragmentation), resulting in severe injuries to the hands and possibly the face and chest. Pattern 1 victims suffer one or more traumatic amputations of the lower limb, whereas pattern 2 victims have fragment wounds scattered over the body. Pattern 2 victims may have fragment injuries of the lower limbs but the damage is not severe enough to cause traumatic amputation.

For peer review only - http://bmjopen.bmj.com/site/about/guidelines.xhtml

This distinguishing feature of a traumatic amputation between pattern 1 and pattern 2 injuries was confirmed in a later report of 4616 APM victims which present further evidence of the severity of injury particularly to the limbs.[3] Of 1077 (23%) pattern 1 victims, 606 (56%) had below knee amputation (BKA), 26 (2%) bilateral BKA, 349 (32%) unilateral above the knee amputations (AKA), 57 (5%) bilateral AKA, and 39 (4%) a combination of AKA and BKA.[3] Their description of the typical pattern 1 injury profile caused by an APM, which has been confirmed by other studies, is of a traumatic amputation of the foot or leg with scattered penetrating injuries elsewhere. [2-5]

The improvised explosive device (IED) is increasingly used in modern conflicts including Afghanistan.[6,7] The majority of these weapons have been directed against pedestrian individuals in a similar fashion to APM.[8,9] As with APM, injuries suffered by victims of the anti-personnel IED (AP-IED) depend on whether they were the target of the explosive device or at some distance from the centre of the explosion. It does not depend on the manufacture of the explosive device, industrial as in APM versus improvised as in AP-IED. Indeed elements of conventional explosives may be used to construct an AP-IED. The blast injury depends on the energy transferred by the explosion to the victim. However it was our impression that pattern 1 injuries caused by AP-IED were significantly worse than those reported for APM.[2] The purpose of this paper is to describe the profile of pattern 1 injury suffered by the target of the AP-IED compared to the target of APM.

For peer review only - http://bmjopen.bmj.com/site/about/guidelines.xhtml

METHODS

The cohort of patients under study was defined as those pedestrian victims of an AP-IED who suffered a traumatic amputation. In order to be included in the study, casualties were required to have been reported by the first responder as being the dismounted (pedestrian) victim of an IED explosion and to have suffered a traumatic amputation. Casualties that were non-pedestrian (i.e. in a vehicle) or whose situation was unknown were excluded. The study was designed to describe the injuries suffered by 100 consecutive targets of AP-IED attacks who presented to the North Atlantic Treaty Organization Role 3 Multinational Medical Unit (R3-MMU) in Kandahar Air Field (KAF). The hospital received casualties from the point of injury or via a forward treatment centre.[10] A R3-MMU is the highest level of in-theatre medical care and would be equivalent to a civilian level-II trauma centre, as defined by the American College of Surgeons-Committee On Trauma. A specifically designated trauma nurse (M.D.), and attending surgeons (J.T., V.M.) prospectively collected data for each patient. Data was collected between January 2010 and July 2011, or until data from 100 victims had been obtained. Data collected included: nationality, age, gender, mortality status, specific injuries, and imaging results. The level of traumatic amputation, the level of soft tissue injury, the fracture pattern (including pelvic fractures) as well as perineal and gluteal injuries were specifically recorded. Generally, coalition patients were transferred after damage control surgery, whereas local patients also received definitive care at the R3-MMU. Data were collected until the time of first discharge from the hospital. In order to minimize bias, patients were included sequentially as a consecutive series with a predefined definition of AP-IED target. The R3-MMU was the only surgical facility in the region and would receive all

For peer review only - http://bmjopen.bmj.com/site/about/guidelines.xhtml

AP-IED victims for care. Patient level data is reported identifying the injuries of each of the 100 victims. The paediatric victims are also presented separately. The data collection was complete with injury data captured for all 100 victims. Categorical data was analysed using the chi-square test. AP-IED data was compared to previously published APM data[2]. This study was approved by the commander of the R3-MMU KAF and by the Research Ethics Board of the University of Western Ontario (REB # 104124).

RESULTS

One hundred consecutive casualties with amputations from AP-IEDs were identified and their injuries were described. All of the patients were male. The mean, and median, age of the victims was 25 years. The age range was from ages 6 to 44 years. There were 9 patients under the age of 18 (Figure 1). Twenty-seven patients were Afghan local nationals and 61 were coalition soldiers (USA, Canada, UK); there were 12 victims whose nationality was not captured (Table 1). Eleven patients were dead upon arrival and another 8 died of their wounds in the hospital, giving a 19% casualty fatality rate. Gender, injury pattern, age or approximate age, was recorded for all victims.

AP-IED victims were more likely than a similar cohort of APM victims to suffer multiple amputations (70.0% vs. 10.4%; $p<0.001$) or genital injury (26.0% vs. 13.4%; $p=0.007$). Seventy victims (70%) sustained multiple amputations: 5 patients suffered quadruple amputations, 27 had triple amputations, and 38 victims had double amputations (Figure 2). Two victims had hip disarticulations, 41 had at least one AKA, and 56 had at least one BKA (including through knee and through ankle amputations). Sixty-five victims had

For peer review only - http://bmjopen.bmj.com/site/about/guidelines.xhtml

bilateral lower extremity amputations and 37 had at least one upper extremity amputation (at the level of the hand or higher) (Table 2). Pelvic fractures were present in 21 victims, all but one of whom had multiple amputations. Forty-six patients had perineal and gluteal injuries; these included 8 anorectal injuries, 8 penile injuries, and 26 scrotal injuries including 10 orchiectomies. There were 13 facial injuries, 9 skull fractures and 3 traumatic brain injuries. Eye injuries were found in 11 patients, none of whom were wearing eye protection. Of the 9 pediatric patients, 3 had triple amputations and 5 had double amputations. There was one pelvic fracture and two perineal injuries among the pediatric victims (Table 3). Pelvic fracture was more likely if the victim had multiple amputations (28.6% vs. 3.3%; p=0.005) or had a perineal or gluteal injury (32.6% vs. 11.1%; p=0.009). Those victims with multiple amputations also had more perineal and gluteal injuries (52.9% vs. 30%; p=0.036) and a higher mortality (24.3% vs. 6.7%; p=0.039).

Therefore, the typical injury profile suffered by targeted victims of AP-IEDs included: bilateral lower extremity amputations (often above the knee); mangling or amputation of an upper extremity; extensive soft tissue injury with deep contamination by soil, extending into gluteal and perineal regions; pelvic ring disruption and genital mutilation.

DISCUSSION

The mechanism of injury is the same for all anti-personnel explosive devices. The severity of injury depends upon the energy transferred by the explosion to the victim. There is nothing inherently different between the APM and the AP-IED. Indeed the AP-

For peer review only - http://bmjopen.bmj.com/site/about/guidelines.xhtml

IED might be considered a subset of APM. However the comparison in this study is between the conventional blast APM, industrially manufactured for national armies and the AP-IED, that were manufactured locally from available materials and used in the recent conflict in Afghanistan. Coupland and Korver believed they were dealing with the former device.[2,3,4] In a textbook reflecting on the Russian experience in Afghanistan, Bruysov and colleagues illustrated the mechanism of injury from the conventional blast APM in a series of experiments using rapid sequence photography which confirmed Coupland and Korver's description of a foot amputation with scattered penetrating injuries elsewhere.[11]

The AP-IED, sometimes portrayed as a primitive or crude weapon crafted from locally available resources because of a lack of access to conventional weapons, has evolved with use in different conflicts. From rudimentary devices of nails in wooden boxes used in Cambodia and Columbia, AP-IEDs used in recent conflicts are better directed and more destructive. The injury profile from the modern AP-IED is far worse than originally described for conventional blast APMs. Whereas the blast APM typically results in a unilateral lower limb amputation, the modern AP-IED causes bilateral high amputation of the lower extremities. Triple amputations were not seen with APM but occurred in 27% of victims of AP-IEDs. Severe perineal and gluteal injury with soft tissue contamination with soil was commonly present in the victims of the AP-IED but is not seen among the ICRC experience with APM injured patients. [2] The energy transfer endured by targeted victims of AP-IEDs must be far greater than that caused by APMs. A measure of the force is the fact that it is sufficiently powerful to disrupt the pelvic ring in one fifth of the

For peer review only - http://bmjopen.bmj.com/site/about/guidelines.xhtml

patients (Figure 3).

It is not the purpose of this paper to undermine the abhorrence of injuries caused by conventional APMs, which are often devastating and ruin lives. Both the APM and AP-IED injure indiscriminately. In this series, almost one in ten victims of AP-IEDs was a child, a tragic feature in common with APMs. Even more disconcerting is the frequency with which children suffer the severest injuries from the powerful explosive force of the AP-IED: 89% suffered multiple amputations and 33% lost three limbs.

The AP-IED victims wore different types of personal protective equipment (PPE) or none at all. Coalition soldiers wore PPE including helmets, body armour, and anti-ballistic eyewear. Each nation issued its own style and brand of PPE. Local Afghan soldiers often used helmets but their use of body armour was more inconsistent and almost never used ballistic eyewear. Civilian casualties were not wearing any PPE. This heterogeneity of PPE use in our sample may have affected the pattern of injury characterized in this study but the sample size was insufficient to determine accurately the injury preventative role of PPE. We felt there was a lower than expected rate of abdominal, thoracic and eye injuries in victims wearing PPE. We also felt that the design purpose of PPE, to prevent fragment injury, was confirmed in patients with pattern 2 injuries who are not included in this study.

It is possible that we have magnified the severity of the injury pattern by concentrating our focus upon patients that met our definition of being targeted by the AP-IED, that is having sustained at least one limb amputation. It is also possible that if a casualty was

For peer review only - http://bmjopen.bmj.com/site/about/guidelines.xhtml

injured by an unexploded ordinance or classical land mine but reported by first responders to have been injured by an AP-IED they may have been erroneously included. However our findings are in keeping with other descriptions of IED injuries in the literature. Jacobs *et al*. classified the limb injury pattern of 103 consecutive casualties of IEDs treated at a UK Role 3 facility in Helmand Province, Afghanistan.[12] They included victims who did not have amputation of a limb. They found 76 victims suffered significant bilateral lower limb injuries, with 50 who required bilateral lower limb amputation. Thirty-three victims suffered genital or perineal injury, 9 sustained pelvic ring disruption, and 40 sustained significant upper limb injury. They found that all pelvic fractures and 80% of genital injuries were sustained among casualties with bilateral lower limb amputations. A retrospective review of the UK Joint Theatre Trauma Registry examined UK services personnel who were casualties of IEDs in Afghanistan, who sustained lower extremity amputations between January 2007 and December 2010.[13] This registry includes post-mortem reports of soldiers who were killed and never received hospital care. The registry is restricted to UK services members and contains no local nationals or civilian injuries. They examined 656 IED victims with 138 killed in action (21%) and 31 who died of their wounds (4.7%) resulting in a 25.9% casualty fatality rate. Of the 169 victims who sustained a traumatic lower extremity amputation, 69 were killed in action (40.8%) and 31 died of their wounds (18.3%). They found that the level of amputation was inversely correlated with survival with only two survivors from hindquarter amputation out of 39 patients with this level of injury. Another retrospective review of the UK Military Trauma Registry focused on soldiers from Afghanistan with bilateral leg amputation.[8] They examined 43 casualties with at least two amputations;

For peer review only - http://bmjopen.bmj.com/site/about/guidelines.xhtml

80% of these were from AP-IEDs with the other 20% being from anti-vehicle-IEDs. The most common bilateral amputation was a bilateral AKA (58%), and 20% of victims suffered triple amputation. 14% of victims sustained open pelvic fractures and 44% suffered perineal or genital injury. No victims survived the loss of all four limbs.

The soft tissue injury is a particularly difficult problem encountered by those caring for victims of AP-IEDs. The directed explosion forces soil up along soft tissue planes far above the point of entry. This may worsen the level of eventual amputation and it condemns the victim to multiple operations to remove the contamination. Even still, it may leave the victim at the mercy of unusual antibiotic resistant soil organisms such as *Acinetobacter baumannii.*[14]

Knowing the pattern of injury helps responders tailor care. As Jacobs and colleagues have suggested, first responders should apply tourniquets bilaterally even if the victim is not bleeding, as haemorrhage is likely to start once resuscitation restores intravascular volume.[12] While current protocols require medics to apply a neck collar to all victims of IEDs, spinal injury is rare in AP-IED victims despite the magnitude of the force.[9] On the other hand, we recommend that all AP-IED victims with perineal injuries or multiple amputations have pelvic binders placed by the first responders, as both are associated with pelvic fracture. This may reduce the large amount of blood that may be lost silently with pelvic disruption. Multiple surgical teams have to work simultaneously to rescue these patients. Surgeons have to be adept at dealing with pelvic and gluteal bleeding, sometimes by pre-peritoneal packing and by ligating the internal iliac artery. Rehabilitation requires mobilization on temporary lower limb prostheses despite injured

upper limbs. Finally, victims of this severe injury pattern need life-long support because disabilities may become more pronounced as the capacity to compensate fades with age.

In collecting information on 100 consecutive patients, we hope to have described a generalizable result. Due to the nature of the conflict and the local culture, all of the patients examined were male. While we would expect the severity of the injury pattern to be generalizable to females, we cannot explicitly determine the effect of the modern AP-IED on the female pelvis and perineum.

The use of conventional APM has decreased in the last 20 years since the campaign to prohibit their use. Pattern 1 injuries seen in recent conflicts are far more often from AP-IEDs than conventional APMs. ICRC includes AP-IEDs in its definition of APM if the victim triggers the weapon. The injury profile described in this report is illustrated in the recent edition of its textbook on war surgery.[15] Despite the huge force endured by the targeted victim of the AP-IED, the casualty fatality rate that we observed was 19%. This rate is an underestimate, as some casualties who were killed at the site of the AP-IED may not have been brought to our facility and others may have died as a result of injuries after transfer or discharge. Reports regarding APM are also affected by this problem. The observed mortality rate with AP-IED is of the same order than that elsewhere.[15] The injury pattern suffered by the target of the AP-IED is markedly worse than that of conventional APM. Pelvic binders and tourniquets should be applied at the point of injury to patients with multiple amputations or perineal injuries. The AP-IED causes more severe injury than APM. It is a weapon which of its nature causes superfluous injury and unnecessary suffering. Just as the reports by Coupland and Korver provided the medical

For peer review only - http://bmjopen.bmj.com/site/about/guidelines.xhtml

evidence upon which the prohibition of conventional APM was based, it is hoped that reports regarding the pattern of injury caused by the modern AP-IED will result in an abhorrence of this weapon and those who use it.

For peer review only

For peer review only - http://bmjopen.bmj.com/site/about/guidelines.xhtml

REFERENCES

1. United Nations. Convention on the Prohibition of the Use, Stockpiling, Production and Transfer of Anti-Personnel Mines and on their Destruction. *United Nations, Treaty Series , vol 2056, p 211.* 1999.

2. Coupland RM, Korver A. Injuries from antipersonnel mines: the experience of the International Committee of the Red Cross. *BMJ* 1991;303(6816):1509-1512.

3. Korver AJ. Injuries of the lower limbs caused by antipersonnel mines: the experience of the International Committee of the Red Cross. *Injury.* 1996;27(7):477-479.

4. Coupland RM. Amputation for antipersonnel mine injuries of the leg: preservation of the tibial stump using a medial gastrocnemius myoplasty. *Annals of the Royal College of Surgeons of England.* 1989;71(6):405-408.

5. Soroush A, Falahati F, Zargar M, Soroush M, Khateri S, Khaji A. Amputations due to landmine and unexploded ordinances in post-war Iran. *Archives of Iranian Medicine.* 2008;11(6):595-597.

6. Jacobs N, Taylor DM, Parker PJ. Changes in surgical workload at the JF Med Gp Role 3 Hospital, Camp Bastion, Afghanistan, November 2008-November 2010. *Injury.* 2012;43(7):1037-1040.

7. Beckett A, Pelletier P, Mamczak C, Benfield R, Elster E. Multidisciplinary trauma team care in Kandahar, Afghanistan: current injury patterns and care practices. *Injury.* 2012;43(12):2072-2077.

For peer review only - http://bmjopen.bmj.com/site/about/guidelines.xhtml

8. Penn-Barwell JG, Bennett PM, Kay A, Sargeant ID. Acute bilateral leg amputation following combat injury in UK servicemen. *Injury.* 2014;45(7):1105-1110.

9. Taddeo J, Devine M, McAlister VC. Cervical spine injury in dismounted improvised explosive device trauma. *Can J Surg.* 2015;58(3 Suppl 3):S104-107.

10. Brisebois R, Hennecke P, Kao R, et al. The Role 3 Multinational Medical Unit at Kandahar Airfield 2005-2010. *Can J Surg.* 2011;54(6):S124-129.

11. Bryusov PG, Shapovalov VM, Artemyev AA, Dulayev AK, Gololobov VG. Combat Injuries of Extremities. Moscow: Military Medical Academy, GEOTAR; 1996. [Translation by ICRC Delegation, Moscow]

12. Jacobs N, Rourke K, Rutherford J, et al. Lower limb injuries caused by improvised explosive devices: proposed 'Bastion classification' and prospective validation. *Injury.* 2014;45(9):1422-1428.

13. Morrison JJ, Hunt N, Midwinter M, Jansen J. Associated injuries in casualties with traumatic lower extremity amputations caused by improvised explosive devices. *The British Journal of Surgery.* 2012;99(3):362-366.

14. O'Shea MK. Acinetobacter in modern warfare. *Int J Antimicrob Agents.* 2012;39(5):363-375.

15. Giannou C, Baldan M, Molde A. War Surgery: working with limited resources in armed conflict and other situations of violence (Volume 2). Geneva: International Committee of the Red Cross; 2103. <https://www.icrc.org/eng/assets/files/publications/icrc-002-4105.pdf>

For peer review only - http://bmjopen.bmj.com/site/about/guidelines.xhtml

Table 1: Victim characteristics

Characteristic	
Number of patients	100
Age, mean (SD)	25 (6.8) yrs
Median	25 yrs
Range	6 – 44 yrs
Male	100
Female	0
Nationality	
Afghan	27
Coalition	61
American	49
Canadian	8
British	4
Not specified	12
Mortality	19
Killed in Action	11
Died of wounds	8

SD = standard deviation

For peer review only - http://bmjopen.bmj.com/site/about/guidelines.xhtml

Table 2: Distribution of amputations

Amputation Type	Overall	Pelvic Fracture	Perineal, Gluteal, Genital Injury	Killed in Action	Died of Wounds	Casualty Fatality Rate
HipDis/TKA/UEA	1	0	0	0	1	1/1
HipDis/-	1	0	0	0	1	1/1
AKA/AKA/UEA/UEA	4	0	3	0	0	0/4
AKA/AKA/UEA	7	3	6	0	0	0/7
AKA/AKA	8	2	4	2	1	3/8
AKA/BKA/UEA	2	1	0	1	1	2/2
AKA/TKA/UEA	1	1	0	0	1	1/1
AKA/BKA	7	1	5	0	0	0/7
AKA/TKA	7	4	6	0	0	0/7
AKA/UEA/UEA	1	0	0	0	0	0/1
AKA/UEA	1	0	0	0	0	0/1
AKA/TAA	1	0	0	0	0	0/1
AKA/-	2	0	0	0	0	0/2
BKA/BKA/UEA/UEA	1	0	0	0	1	1/1
BKA/BKA/UEA	13	4	6	4	2	6/13
BKA/TKA/UEA	1	0	1	0	0	0/1
BKA/BKA	9	3	5	2	0	2/9
BKA/UEA	2	0	0	0	0	0/2
BKA/TKA	1	0	0	1	0	1/1
BKA/-	22	1	9	1	0	1/22
TKA/TKA/UEA	1	1	1	0	0	0/1
TKA/TKA	1	0	0	0	0	0/1
TKA/-	1	0	0	0	0	0/1
TAA/UEA	1	0	0	0	0	0/1
TAA/-	3	0	0	0	0	0/3
UEA/-	1	0	0	0	0	0/1

HipDis, hip disarticulation; AKA, above knee amputation; BKA, below knee amputation; TKA, though knee amputation, TAA, though ankle amputation; UEA, upper extremity amputation, which includes above elbow, below elbow and hand amputations.

Table 3: Distribution of pediatric amputations

Age	Amputation Type	Pelvic Fracture	Perineal, Gluteal, Genital Injury	Disposition
16	AKA/BKA	No	No	Survived
14	AKA/AKA	No	Yes	DOW
14	AKA/UEA/UEA	No	No	Survived
11	UEA/TKA/AKA	Yes	No	DOW
10	BKA/BKA/UEA	No	No	KIA
10	UEA/TAA	No	No	Survived
10	BKA/AKA	No	Yes	Survived
10	BKA/BKA	No	No	KIA
6	UEA	No	No	Survived

AKA, above knee amputation; BKA, below knee amputation; TKA, though knee amputation, TAA, though ankle amputation; UEA, upper extremity amputation, which includes above elbow, below elbow and hand amputations; DOW, died of wounds; KIA, killed in action.

For peer review only - http://bmjopen.bmj.com/site/about/guidelines.xhtml

Legend for figures

Figure 1: Age ranges of AP-IED targeted victims

Figure 2: Numbers of traumatic limb amputations per targeted victim of the AP-IED

Figure 3: X-ray taken for placement of a pelvic external fixator showing: disruption of pelvic ring by the force of an anti-personnel improvised explosive device; the arrow indicates combat gauze packing a severe perineal injury; silica laden soil injection by the explosion.

For peer review only

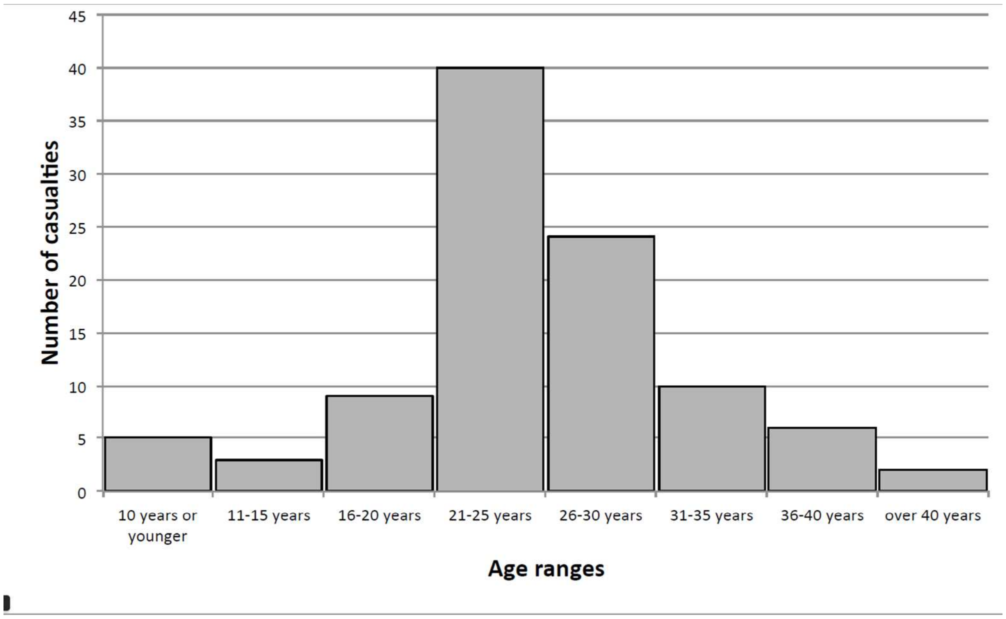

Figure 1: Age ranges of AP-IED targeted victims

125x76mm (300 x 300 DPI)

For peer review only - http://bmjopen.bmj.com/site/about/guidelines.xhtml

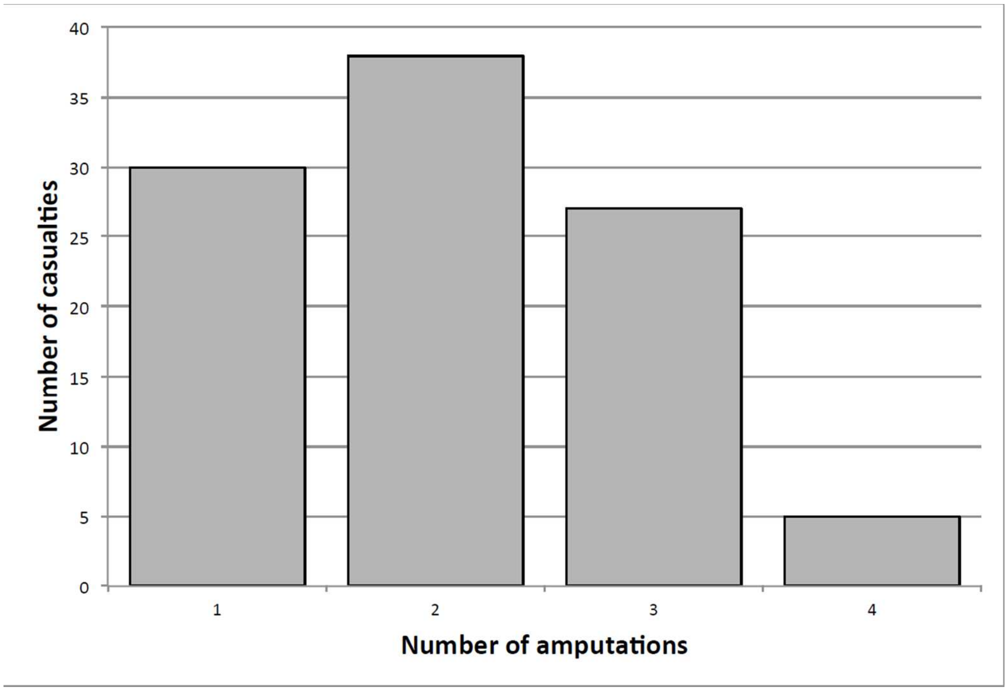

Figure 2: Numbers of traumatic limb amputations per targeted victim of the AP-IED

112x76mm (300 x 300 DPI)

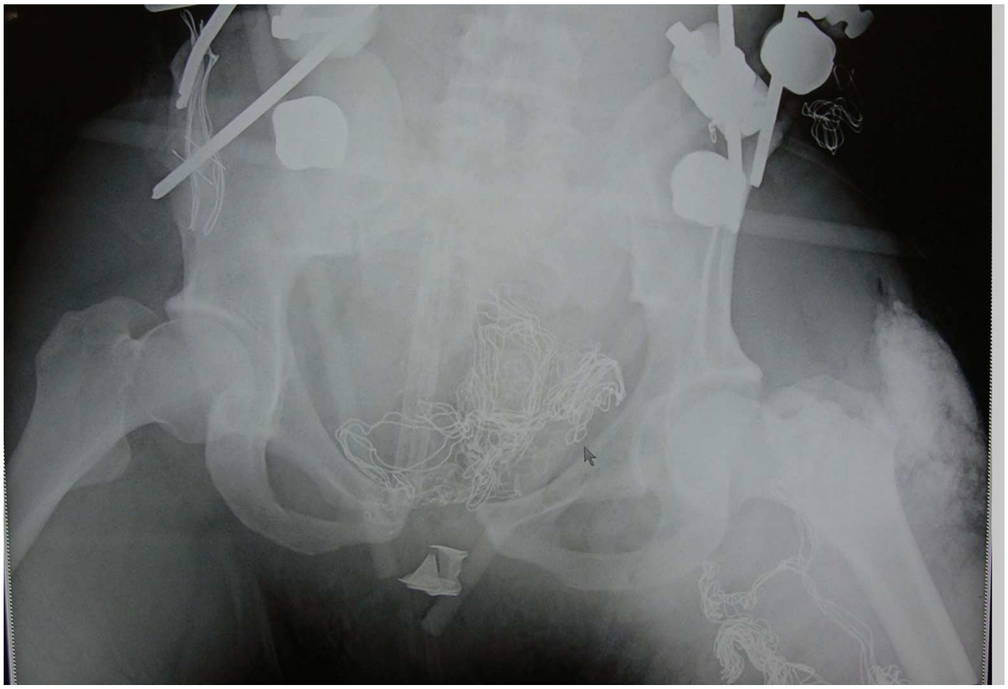

Figure 3: X-ray taken for placement of a pelvic external fixator showing: disruption of pelvic ring by the force of an anti-personnel improvised explosive device; the arrow indicates combat gauze packing a severe perineal injury; silica laden soil injection by the explosion.

440x300mm (300 x 300 DPI)

review only

STROBE Statement—checklist of items that should be included in reports of observational studies

	Item No	Recommendation
**Title and abstract**	1	(*a*) Indicate the study's design with a commonly used term in the title or the abstract **Pg 4**
		(*b*) Provide in the abstract an informative and balanced summary of what was done and what was found **Pg 4**
**Introduction**		
Background/rationale	2	Explain the scientific background and rationale for the investigation being reported **Pg 7-8**
Objectives	3	State specific objectives, including any prespecified hypotheses **Pg 9**
**Methods**		
Study design	4	Present key elements of study design early in the paper **Pg 9**
Setting	5	Describe the setting, locations, and relevant dates, including periods of recruitment, exposure, follow-up, and data collection **Pg 9**
Participants	6	(*a*) *Cohort study*—Give the eligibility criteria, and the sources and methods of selection of participants. Describe methods of follow-up **Pg 9** *Case-control study*—Give the eligibility criteria, and the sources and methods of case ascertainment and control selection. Give the rationale for the choice of cases and controls *Cross-sectional study*—Give the eligibility criteria, and the sources and methods of selection of participants
		(*b*) *Cohort study*—For matched studies, give matching criteria and number of exposed and unexposed N/A *Case-control study*—For matched studies, give matching criteria and the number of controls per case
Variables	7	Clearly define all outcomes, exposures, predictors, potential confounders, and effect modifiers. Give diagnostic criteria, if applicable **Pg 9**
Data sources/ measurement	8*	For each variable of interest, give sources of data and details of methods of assessment (measurement). Describe comparability of assessment methods if there is more than one group **Pg 9**
Bias	9	Describe any efforts to address potential sources of bias **Pg 10**
Study size	10	Explain how the study size was arrived at **Pg 9**
Quantitative variables	11	Explain how quantitative variables were handled in the analyses. If applicable, describe which groupings were chosen and why **N/A**
Statistical methods	12	(*a*) Describe all statistical methods, including those used to control for confounding **Pg 10**
		(*b*) Describe any methods used to examine subgroups and interactions **Pg 10**
		(*c*) Explain how missing data were addressed **Pg 10**
		(*d*) *Cohort study*—If applicable, explain how loss to follow-up was addressed *Case-control study*—If applicable, explain how matching of cases and controls was addressed *Cross-sectional study*—If applicable, describe analytical methods taking account of sampling strategy
		(*e*) Describe any sensitivity analyses

Continued on next page

For peer review only - http://bmjopen.bmj.com/site/about/guidelines.xhtml

**Results**		
Participants	13*	(a) Report numbers of individuals at each stage of study—eg numbers potentially eligible, examined for eligibility, confirmed eligible, included in the study, completing follow-up, and analysed **Pg 10**
		(b) Give reasons for non-participation at each stage
		(c) Consider use of a flow diagram
Descriptive data	14*	(a) Give characteristics of study participants (eg demographic, clinical, social) and information on exposures and potential confounders **Pg 10**
		(b) Indicate number of participants with missing data for each variable of interest **Pg 10 – Table 1**
		(c) *Cohort study*—Summarise follow-up time (eg, average and total amount)
Outcome data	15*	*Cohort study*—Report numbers of outcome events or summary measures over time
		Case-control study—Report numbers in each exposure category, or summary measures of exposure
		Cross-sectional study—Report numbers of outcome events or summary measures
Main results	16	(*a*) Give unadjusted estimates and, if applicable, confounder-adjusted estimates and their precision (eg, 95% confidence interval). Make clear which confounders were adjusted for and why they were included **Pg 10-11**
		(*b*) Report category boundaries when continuous variables were categorized
		(*c*) If relevant, consider translating estimates of relative risk into absolute risk for a meaningful time period
Other analyses	17	Report other analyses done—eg analyses of subgroups and interactions, and sensitivity analyses
**Discussion**		
Key results	18	Summarise key results with reference to study objectives **Pg 12**
Limitations	19	Discuss limitations of the study, taking into account sources of potential bias or imprecision. Discuss both direction and magnitude of any potential bias **Pg 12**
Interpretation	20	Give a cautious overall interpretation of results considering objectives, limitations, multiplicity of analyses, results from similar studies, and other relevant evidence **Pg 13-14**
Generalisability	21	Discuss the generalisability (external validity) of the study results **Pg 15**
**Other information**		
Funding	22	Give the source of funding and the role of the funders for the present study and, if applicable, for the original study on which the present article is based **Pg 3**

*Give information separately for cases and controls in case-control studies and, if applicable, for exposed and unexposed groups in cohort and cross-sectional studies.

Note: An Explanation and Elaboration article discusses each checklist item and gives methodological background and published examples of transparent reporting. The STROBE checklist is best used in conjunction with this article (freely available on the Web sites of PLoS Medicine at http://www.plosmedicine.org/, Annals of Internal Medicine at http://www.annals.org/, and Epidemiology at http://www.epidem.com/). Information on the STROBE Initiative is available at www.strobe-statement.org.

For peer review only

BMJ Open

Injury profile suffered by targets of antipersonnel improvised explosive devices: a prospective cohort study

Journal:	*BMJ Open*
Manuscript ID	bmjopen-2016-014697.R3
Article Type:	Research
Date Submitted by the Author:	15-Jun-2017
Complete List of Authors:	Smith, Shane; Royal Canadian Medical Service; Western University, Surgery<br>Devine, Melissa ; Royal Canadian Medical Service, Critical Care Nursing<br>Taddeo, Joseph; Maine Veterans' Affairs Medical Center, Surgery<br>McAlister, Vivian; Royal Canadian Medical Service, ; Western University, Surgery
**Primary Subject Heading**:	Global health
Secondary Subject Heading:	Emergency medicine, Surgery
Keywords:	blast injury, war, amputation, soft tissue injury, landmines

SCHOLARONE™
Manuscripts

For peer review only

Injury profile suffered by targets of antipersonnel improvised explosive devices: a prospective cohort study

Shane Smith[1,2]

Melissa Devine[2]

Joseph Taddeo[3]

Vivian McAlister[1,2*]

[1]Royal Canadian Medical Service

[2]Division of General Surgery, University of Western Ontario, Ontario, Canada

[3]Maine Veterans' Affairs Medical Center, Augusta, Maryland, USA

Shane Smith

Medical Officer, Royal Canadian Medical Service

C4-211 University Hospital, London, Ontario, Canada, N6A 5A5

Melissa Devine

Critical Care Nursing Officer, Royal Canadian Medical Service

QEII Health Science Centre, 1276 South Park Street 3rd Floor Bethune, Room 371

Halifax, NS B3H 2Y9

Joseph Taddeo

General-Trauma Surgeon, Maine Veterans' Affairs Medical Center

For peer review only - http://bmjopen.bmj.com/site/about/guidelines.xhtml

For peer review only

1 VA Center Augusta, ME 04330, USA

Vivian McAlister

General Surgeon, Royal Canadian Medial Service

C8-005 University Hospital, London, Ontario, Canada, N6A 5A5

Telephone: (519) 663-2920, Fax: 1-(855)-538-2926

Email: *vmcalist@uwo.ca*

***Corresponding author**

Word count: 2,809

Keywords: Improvised Explosive Device; IED; Blast; Amputation; Trauma; Military;

For peer review only - http://bmjopen.bmj.com/site/about/guidelines.xhtml

The views expressed in this paper are those of the authors and do not constitute the views or policies of the Canadian Armed Forces or the United States Navy.

Competing interests: All authors have completed the ICMJE uniform disclosure form at www.icmje.org/coi_disclosure.pdf and declare: no support from any organisation for the submitted work; no financial relationships with any organisations that might have an interest in the submitted work in the previous three years; no other relationships or activities that could appear to have influenced the submitted work.

Contributions: S.S. analysed the data and drafted the manuscript. M.D. conducted data collection. V.M. and J.T. were the treating trauma surgeons of the casualties and designers of the study. They also conducted data collection. The manuscript has been reviewed and approved by all authors. All authors had full access to all of the data in the study and can take responsibility for the integrity of the data and the accuracy of the data analysis.

This study did not receive any outside funding. The Canadian Armed Forces employed S.S., M.D., and V.M. at the time of the study. The United States Navy employed J.D. at the time of the study.

Transparency declaration: The lead author affirms that the manuscript is an honest, accurate, and transparent account of the study being reported; that no important aspects of the study have been omitted; and that any discrepancies from the study as planned have been explained.

For peer review only - http://bmjopen.bmj.com/site/about/guidelines.xhtml

ABSTRACT

Objective: To describe Pattern 1 injuries caused by the antipersonnel improvised explosive device (AP-IED) in comparison to those previously for anti-personnel mines (APM)

Design: Prospective cohort study of 100 consecutive pedestrian victims of an AP-IED, with traumatic amputation without regard for gender, nationality or military status.

Setting: Multinational Medical Unit at Kandahar Air Field, Afghanistan.

Participants: One hundred consecutive patients, all male, 6 to 44 years old.

Main outcome measures: The details of injuries were recorded in order to describe the pattern and characterize the injuries suffered by the target of AP-IEDs. The level of amputation, the level of soft tissue injury, the fracture pattern (including pelvic fractures) as well as perineal, gluteal, genital, and other injuries were recorded.

Results: Victims of AP-IED were more likely, compared to APM victims, to have multiple amputations (70.0% vs. 10.4%; $p<0.001$) or genital injury (26% vs. 13%; $p=0.007$). Multiple amputations occurred in 70 patients: 5 quadruple amputations, 27 triple amputations, and 38 double amputations. Pelvic fracture occurred in 21 victims, all but one of whom had multiple amputations. Severe perineal, gluteal, or genital injuries were present in 46 patients. Severe soft tissue injury was universal, with injection of contaminated soil along tissue planes well above entry sites. There were 13 facial injuries, 9 skull fractures, and 3 traumatic brain injuries. Eleven eye injuries were seen; none of the victims with eye injuries were wearing eye protection. The casualty fatality rate was at least 19%. The presence of more than one amputation was associated with a

For peer review only - http://bmjopen.bmj.com/site/about/guidelines.xhtml

For peer review only

higher rate of pelvic fracture (28.6% vs. 3.3%; p=0.005) and perineal - gluteal injury (32.6% vs. 11.1%; p=0.009).

Conclusion: The injury pattern suffered by the target of the AP-IED is markedly worse than that of conventional APM. Pelvic binders and tourniquets should be applied at the point of injury to patients with multiple amputations or perineal injuries.

For peer review only

For peer review only - http://bmjopen.bmj.com/site/about/guidelines.xhtml

ARTICLE SUMMARY

Strengths and limitations of this study:

This is a prospective consecutive series with a predefined definition of the target of the AP-IED attack used as inclusion criteria.

The definition of the target of the AP-IED was chosen so as to facilitate comparison to the International Committee of the Red Cross land mine data. This excluded injured bystanders who may have more minor injuries.

The Role 3 Multinational Medical Unit was the only surgical facility in the region. This maximized catchment of AP-IED victims and minimized selection bias.

Patients who died at the scene of the explosion may not have been evacuated to the Role 3 Multinational Medical Unit and victims who died after discharge would not have been included in this study. This may have resulted in an underestimation of the AP-IED's lethality. However studies of APM victims, to which this study is compared, are subject to a similar risk of underestimation.

For peer review only - http://bmjopen.bmj.com/site/about/guidelines.xhtml

INTRODUCTION

Antipersonnel mines (APM) came into widespread use in the Second World War. Originally they were used to protect anti-tank mines, but then went on to become a weapon system in and of themselves. They were designed to injure but not kill, in order to remove a target from combat and to increase the logistical burden of caring for the casualty. These weapons are typically buried and left for the target to trigger. The weapon is indiscriminate because non-combatants such as children and civilians may detonate them. They are also frequently left buried and active after fighting in that region, or the conflict itself, has ended. Following a public relations campaign that highlighted the indiscriminate injuries cause by APMs, 162 countries signed the 1997 Ottawa treaty promising to cease their production and use.[1] An important basis of that campaign was the clinical description of APM related injuries by Robin Coupland and Adriaan Korver for the International Committee of the Red Cross (ICRC).[2] Three patterns of injury were found among 754 victims treated at two ICRC hospitals following wounding by blast or fragmentation mines. In pattern 1 injuries, the victim triggers a blast mine by stepping on it and suffers the full effect of the explosion. In pattern 2 injuries, victims are farther away from the centre of the explosion, whether it is a blast or fragmentation mine, and suffer wounds from fragments. Pattern 3 injuries are caused by handling the mine (blast or fragmentation), resulting in severe injuries to the hands and possibly the face and chest. Pattern 1 victims suffer one or more traumatic amputations of the lower limb, whereas pattern 2 victims have fragment wounds scattered over the body and only rarely suffer amputation.

For peer review only - http://bmjopen.bmj.com/site/about/guidelines.xhtml

This distinguishing feature of a traumatic amputation between pattern 1 and pattern 2 injuries was confirmed in a later report of 4616 APM victims which present further evidence of the severity of injury particularly to the limbs.[3] Of 1077 (23%) pattern 1 victims, 606 (56%) had below knee amputation (BKA), 26 (2%) bilateral BKA, 349 (32%) unilateral above the knee amputations (AKA), 57 (5%) bilateral AKA, and 39 (4%) a combination of AKA and BKA.[3] Their description of the typical pattern 1 injury profile caused by an APM, which has been confirmed by other studies, is of a traumatic amputation of the foot or leg with scattered penetrating injuries elsewhere. [2-5]

The improvised explosive device (IED) is increasingly used in modern conflicts including Afghanistan.[6,7] The majority of these weapons have been directed against pedestrian individuals in a similar fashion to APM.[8,9] As with APM, injuries suffered by victims of the anti-personnel IED (AP-IED) depend on whether they were the target of the explosive device or at some distance from the centre of the explosion. It does not depend on the manufacture of the explosive device, industrial as in APM versus improvised as in AP-IED. Indeed elements of conventional explosives may be used to construct an AP-IED. The blast injury depends on the energy transferred by the explosion to the victim. However it was our impression that pattern 1 injuries caused by AP-IED were significantly worse than those reported for APM.[2] The purpose of this paper is to describe the profile of pattern 1 injury suffered by the target of the AP-IED compared to the target of APM.

For peer review only - http://bmjopen.bmj.com/site/about/guidelines.xhtml

METHODS

The cohort of patients under study was defined as those pedestrian victims of an AP-IED who suffered a traumatic amputation. In order to be included in the study, casualties were required to have been reported by the first responder as being the dismounted (pedestrian) victim of an IED explosion and to have suffered a traumatic amputation. Casualties that were non-pedestrian (i.e. in a vehicle) or whose situation was unknown were excluded. The study was designed to describe the injuries suffered by 100 consecutive targets of AP-IED attacks who presented to the North Atlantic Treaty Organization Role 3 Multinational Medical Unit (R3-MMU) in Kandahar Air Field (KAF). The hospital received casualties from the point of injury or via a forward treatment centre.[10] A R3-MMU is the highest level of in-theatre medical care and would be equivalent to a civilian level-II trauma centre, as defined by the American College of Surgeons-Committee On Trauma. A specifically designated trauma nurse (M.D.), and attending surgeons (J.T., V.M.) prospectively collected data for each patient. Data was collected between January 2010 and July 2011, or until data from 100 victims had been obtained. Data collected included: nationality, age, gender, mortality status, specific injuries, and imaging results. The level of traumatic amputation, the level of soft tissue injury, the fracture pattern (including pelvic fractures) as well as perineal and gluteal injuries were specifically recorded. Generally, coalition patients were transferred after damage control surgery, whereas local patients also received definitive care at the R3-MMU. Data were collected until the time of first discharge from the hospital. In order to minimize bias, patients were included sequentially as a consecutive series with a predefined definition of AP-IED target. The R3-MMU was the only surgical facility in the region and would receive all

For peer review only - http://bmjopen.bmj.com/site/about/guidelines.xhtml

AP-IED victims for care. Patient level data is reported identifying the injuries of each of the 100 victims. The paediatric victims are also presented separately. The data collection was complete with injury data captured for all 100 victims. Categorical data was analysed using the chi-square test. AP-IED data was compared to previously published APM data[2]. This study was approved by the commander of the R3-MMU KAF and by the Research Ethics Board of the University of Western Ontario (REB # 104124).

RESULTS

One hundred consecutive casualties with amputations from AP-IEDs were identified and their injuries were described. All of the patients were male. The mean, and median, age of the victims was 25 years. The age range was from ages 6 to 44 years. There were 9 patients under the age of 18 (Figure 1). Twenty-seven patients were Afghan local nationals and 61 were coalition soldiers (USA, Canada, UK); there were 12 victims whose nationality was not captured (Table 1). Eleven patients were dead upon arrival and another 8 died of their wounds in the hospital, giving a 19% casualty fatality rate. Gender, injury pattern, age or approximate age, was recorded for all victims.

AP-IED victims were more likely than a similar cohort of APM victims to suffer multiple amputations (70.0% vs. 10.4%; $p<0.001$) or genital injury (26.0% vs. 13.4%; $p=0.007$). Seventy victims (70%) sustained multiple amputations: 5 patients suffered quadruple amputations, 27 had triple amputations, and 38 victims had double amputations (Figure 2). Two victims had hip disarticulations, 41 had at least one AKA, and 56 had at least one

For peer review only - http://bmjopen.bmj.com/site/about/guidelines.xhtml

BKA (including through knee and through ankle amputations). Sixty-five victims had bilateral lower extremity amputations and 37 had at least one upper extremity amputation (at the level of the hand or higher) (Table 2). Pelvic fractures were present in 21 victims, all but one of whom had multiple amputations. Forty-six patients had perineal and gluteal injuries; these included 8 anorectal injuries, 8 penile injuries, and 26 scrotal injuries including 10 orchiectomies. There were 13 facial injuries, 9 skull fractures and 3 traumatic brain injuries. Eye injuries were found in 11 patients, none of whom were wearing eye protection. Of the 9 pediatric patients, 3 had triple amputations and 5 had double amputations. There was one pelvic fracture and two perineal injuries among the pediatric victims (Table 3). Pelvic fracture was more likely if the victim had multiple amputations (28.6% vs. 3.3%; p=0.005) or had a perineal or gluteal injury (32.6% vs. 11.1%; p=0.009). Those victims with multiple amputations also had more perineal and gluteal injuries (52.9% vs. 30%; p=0.036) and a higher mortality (24.3% vs. 6.7%; p=0.039).

Therefore, the typical injury profile suffered by targeted victims of AP-IEDs included: bilateral lower extremity amputations (often above the knee); mangling or amputation of an upper extremity; extensive soft tissue injury with deep contamination by soil, extending into gluteal and perineal regions; pelvic ring disruption and genital mutilation.

DISCUSSION

The mechanism of injury is the same for all anti-personnel explosive devices. The severity of injury depends upon the energy transferred by the explosion to the victim. There is nothing inherently different between the APM and the AP-IED. Indeed the AP-

For peer review only - http://bmjopen.bmj.com/site/about/guidelines.xhtml

IED might be considered a subset of APM. However the comparison in this study is between the conventional blast APM, industrially manufactured for national armies and the AP-IED, that were manufactured locally from available materials and used in the recent conflict in Afghanistan. Coupland and Korver believed they were dealing with the former device.[2,3,4] In a textbook reflecting on the Russian experience in Afghanistan, Bruysov and colleagues illustrated the mechanism of injury from the conventional blast APM in a series of experiments using rapid sequence photography which confirmed Coupland and Korver's description of a foot amputation with scattered penetrating injuries elsewhere.[11]

The AP-IED, sometimes portrayed as a primitive or crude weapon crafted from locally available resources because of a lack of access to conventional weapons, has evolved with use in different conflicts. From rudimentary devices of nails in wooden boxes used in Cambodia and Columbia, AP-IEDs used in recent conflicts are better directed and more destructive. The injury profile from the modern AP-IED is far worse than originally described for conventional blast APMs. Whereas the blast APM typically results in a unilateral lower limb amputation, the modern AP-IED causes bilateral high amputation of the lower extremities. Triple amputations were not seen with APM but occurred in 27% of victims of AP-IEDs. Severe perineal and gluteal injury with soft tissue contamination with soil was commonly present in the victims of the AP-IED but is not seen among the ICRC experience with APM injured patients.[2] The energy transfer endured by targeted victims of AP-IEDs must be far greater than that caused by APMs. A measure of the force is the fact that it is sufficiently powerful to disrupt the pelvic ring in one fifth of the

For peer review only - http://bmjopen.bmj.com/site/about/guidelines.xhtml

patients (Figure 3).

It is not the purpose of this paper to undermine the abhorrence of injuries caused by conventional APMs, which are often devastating and ruin lives. Both the APM and AP-IED injure indiscriminately. In this series, almost one in ten victims of AP-IEDs was a child, a tragic feature in common with APMs. Even more disconcerting is the frequency with which children suffer the severest injuries from the powerful explosive force of the AP-IED: 89% suffered multiple amputations and 33% lost three limbs.

The AP-IED victims wore different types of personal protective equipment (PPE) or none at all. Coalition soldiers wore PPE including helmets, body armour, and anti-ballistic eyewear. Each nation issued its own style and brand of PPE. Local Afghan soldiers often used helmets but their use of body armour was more inconsistent and almost never used ballistic eyewear. Civilian casualties were not wearing any PPE. This heterogeneity of PPE use in our sample may have affected the pattern of injury characterized in this study but the sample size was insufficient to determine accurately the injury preventative role of PPE. We felt there was a lower than expected rate of abdominal, thoracic and eye injuries in victims wearing PPE. We also felt that the design purpose of PPE, to prevent fragment injury, was confirmed in patients with pattern 2 injuries who are not included in this study.

It is possible that we have magnified the severity of the injury pattern by concentrating our focus upon patients that met our definition of being targeted by the AP-IED, that is having sustained at least one limb amputation. It is also possible that if a casualty was injured by an unexploded ordinance or classical land mine but reported by first

For peer review only - http://bmjopen.bmj.com/site/about/guidelines.xhtml

responders to have been injured by an AP-IED they may have been erroneously included. However our findings are in keeping with other descriptions of IED injuries in the literature. Jacobs *et al*. classified the limb injury pattern of 103 consecutive casualties of IEDs treated at a UK Role 3 facility in Helmand Province, Afghanistan.[12] They included victims who did not have amputation of a limb. They found 76 victims suffered significant bilateral lower limb injuries, with 50 who required bilateral lower limb amputation. Thirty-three victims suffered genital or perineal injury, 9 sustained pelvic ring disruption, and 40 sustained significant upper limb injury. They found that all pelvic fractures and 80% of genital injuries were sustained among casualties with bilateral lower limb amputations. A retrospective review of the UK Joint Theatre Trauma Registry examined UK services personnel who were casualties of IEDs in Afghanistan, who sustained lower extremity amputations between January 2007 and December 2010.[13] This registry includes post-mortem reports of soldiers who were killed and never received hospital care. The registry is restricted to UK services members and contains no local nationals or civilian injuries. They examined 656 IED victims with 138 killed in action (21%) and 31 who died of their wounds (4.7%) resulting in a 25.9% casualty fatality rate. Of the 169 victims who sustained a traumatic lower extremity amputation, 69 were killed in action (40.8%) and 31 died of their wounds (18.3%). They found that the level of amputation was inversely correlated with survival with only two survivors from hindquarter amputation out of 39 patients with this level of injury. Another retrospective review of the UK Military Trauma Registry focused on soldiers from Afghanistan with bilateral leg amputation.[8] They examined 43 casualties with at least two amputations; 80% of these were from AP-IEDs with the other 20% being from anti-vehicle-IEDs. The

most common bilateral amputation was a bilateral AKA (58%), and 20% of victims suffered triple amputation. 14% of victims sustained open pelvic fractures and 44% suffered perineal or genital injury. No victims survived the loss of all four limbs.

The soft tissue injury is a particularly difficult problem encountered by those caring for victims of AP-IEDs. The directed explosion forces soil up along soft tissue planes far above the point of entry. This may worsen the level of eventual amputation and it condemns the victim to multiple operations to remove the contamination. Even still, it may leave the victim at the mercy of unusual antibiotic resistant soil organisms such as *Acinetobacter baumannii.*[14]

Knowing the pattern of injury helps responders tailor care. As Jacobs and colleagues have suggested, first responders should apply tourniquets bilaterally even if the victim is not bleeding, as haemorrhage is likely to start once resuscitation restores intravascular volume.[12] While current protocols require medics to apply a neck collar to all victims of IEDs, spinal injury is rare in AP-IED victims despite the magnitude of the force.[9] On the other hand, we recommend that all AP-IED victims with perineal injuries or multiple amputations have pelvic binders placed by the first responders, as both are associated with pelvic fracture. This may reduce the large amount of blood that may be lost silently with pelvic disruption. Multiple surgical teams have to work simultaneously to rescue these patients. Surgeons have to be adept at dealing with pelvic and gluteal bleeding, sometimes by pre-peritoneal packing and by ligating the internal iliac artery. Rehabilitation requires mobilization on temporary lower limb prostheses despite injured upper limbs. Finally, victims of this severe injury pattern need life-long support because

For peer review only - http://bmjopen.bmj.com/site/about/guidelines.xhtml

disabilities may become more pronounced as the capacity to compensate fades with age.

In collecting information on 100 consecutive patients, we hope to have described a generalizable result. Due to the nature of the conflict and the local culture, all of the patients examined were male. While we would expect the severity of the injury pattern to be generalizable to females, we cannot explicitly determine the effect of the modern AP-IED on the female pelvis and perineum.

The use of conventional APM has decreased in the last 20 years since the campaign to prohibit their use. Pattern 1 injuries seen in recent conflicts are far more often from AP-IEDs than conventional APMs. ICRC includes AP-IEDs in the category of APM if the victim triggers the weapon. The injury profile illustrated in the most recent edition of its textbook on war surgery includes those injured by conventional landmines and AP-IEDS.[15] Indeed, there may be overlap in the pattern 1 injury profile of each weapon. For instance, perineal injuries are possible with landmines but are very rare. The difference between the weapons is a matter of the force endured by the victim. Pelvic binders and tourniquets should be applied at the point of injury to patients with multiple amputations or perineal injuries.

Despite the huge force endured by the targeted victim of the AP-IED, the survival rate that we observed was over 80% which is comparable to that reported elsewhere.[15] While the case fatality rate does not account for casualties who were killed at the site of the AP-IED but not brought to the facility or patients that died as a result of injuries after transfer or discharge, the survival rate might be considered greater than expected considering the

For peer review only - http://bmjopen.bmj.com/site/about/guidelines.xhtml

force endured. The injury pattern suffered by the survivors of the AP-IED is markedly worse than that of conventional APM. It is a weapon which of its nature causes superfluous injury and unnecessary suffering. Just as the reports by Coupland and Korver provided the medical evidence upon which the prohibition of conventional APM was based, it is hoped that reports regarding the pattern of injury caused by the modern AP-IED will result in an abhorrence of this weapon and those who use it.

For peer review only

For peer review only - http://bmjopen.bmj.com/site/about/guidelines.xhtml

REFERENCES

1. United Nations. Convention on the Prohibition of the Use, Stockpiling, Production and Transfer of Anti-Personnel Mines and on their Destruction. *United Nations, Treaty Series , vol 2056, p 211.* 1999.

2. Coupland RM, Korver A. Injuries from antipersonnel mines: the experience of the International Committee of the Red Cross. *BMJ* 1991;303(6816):1509-1512.

3. Korver AJ. Injuries of the lower limbs caused by antipersonnel mines: the experience of the International Committee of the Red Cross. *Injury.* 1996;27(7):477-479.

4. Coupland RM. Amputation for antipersonnel mine injuries of the leg: preservation of the tibial stump using a medial gastrocnemius myoplasty. *Annals of the Royal College of Surgeons of England.* 1989;71(6):405-408.

5. Soroush A, Falahati F, Zargar M, Soroush M, Khateri S, Khaji A. Amputations due to landmine and unexploded ordinances in post-war Iran. *Archives of Iranian Medicine.* 2008;11(6):595-597.

6. Jacobs N, Taylor DM, Parker PJ. Changes in surgical workload at the JF Med Gp Role 3 Hospital, Camp Bastion, Afghanistan, November 2008-November 2010. *Injury.* 2012;43(7):1037-1040.

7. Beckett A, Pelletier P, Mamczak C, Benfield R, Elster E. Multidisciplinary trauma team care in Kandahar, Afghanistan: current injury patterns and care practices. *Injury.* 2012;43(12):2072-2077.

For peer review only - http://bmjopen.bmj.com/site/about/guidelines.xhtml

8. Penn-Barwell JG, Bennett PM, Kay A, Sargeant ID. Acute bilateral leg amputation following combat injury in UK servicemen. *Injury.* 2014;45(7):1105-1110.

9. Taddeo J, Devine M, McAlister VC. Cervical spine injury in dismounted improvised explosive device trauma. *Can J Surg.* 2015;58(3 Suppl 3):S104-107.

10. Brisebois R, Hennecke P, Kao R, et al. The Role 3 Multinational Medical Unit at Kandahar Airfield 2005-2010. *Can J Surg.* 2011;54(6):S124-129.

11. Bryusov PG, Shapovalov VM, Artemyev AA, Dulayev AK, Gololobov VG. Combat Injuries of Extremities. Moscow: Military Medical Academy, GEOTAR; 1996. [Translation by ICRC Delegation, Moscow]

12. Jacobs N, Rourke K, Rutherford J, et al. Lower limb injuries caused by improvised explosive devices: proposed 'Bastion classification' and prospective validation. *Injury.* 2014;45(9):1422-1428.

13. Morrison JJ, Hunt N, Midwinter M, Jansen J. Associated injuries in casualties with traumatic lower extremity amputations caused by improvised explosive devices. *The British Journal of Surgery.* 2012;99(3):362-366.

14. O'Shea MK. Acinetobacter in modern warfare. *Int J Antimicrob Agents.* 2012;39(5):363-375.

15. Giannou C, Baldan M, Molde A. War Surgery: working with limited resources in armed conflict and other situations of violence (Volume 2). Geneva: International Committee of the Red Cross; 2013. <https://www.icrc.org/eng/assets/files/publications/icrc-002-4105.pdf>

For peer review only - http://bmjopen.bmj.com/site/about/guidelines.xhtml

Data sharing: Subject level data will be stored and may be available by contacting the authors.

For peer review only

For peer review only - http://bmjopen.bmj.com/site/about/guidelines.xhtml

.

For peer review only

For peer review only - http://bmjopen.bmj.com/site/about/guidelines.xhtml

Table 1: Victim characteristics

Characteristic	
Number of patients	100
Age, mean (SD)	25 (6.8) yrs
Median	25 yrs
Range	6 – 44 yrs
Male	100
Female	0
Nationality	
Afghan	27
Coalition	61
American	49
Canadian	8
British	4
Not specified	12
Mortality	19
Killed in Action	11
Died of wounds	8

SD = standard deviation

For peer review only

Table 2: Distribution of amputations

Amputation Type	Overall	Pelvic Fracture	Perineal, Gluteal, Genital Injury	Killed in Action	Died of Wounds	Casualty Fatality Rate
HipDis/TKA/UEA	1	0	0	0	1	1/1
HipDis/-	1	0	0	0	1	1/1
AKA/AKA/UEA/UEA	4	0	3	0	0	0/4
AKA/AKA/UEA	7	3	6	0	0	0/7
AKA/AKA	8	2	4	2	1	3/8
AKA/BKA/UEA	2	1	0	1	1	2/2
AKA/TKA/UEA	1	1	0	0	1	1/1
AKA/BKA	7	1	5	0	0	0/7
AKA/TKA	7	4	6	0	0	0/7
AKA/UEA/UEA	1	0	0	0	0	0/1
AKA/UEA	1	0	0	0	0	0/1
AKA/TAA	1	0	0	0	0	0/1
AKA/-	2	0	0	0	0	0/2
BKA/BKA/UEA/UEA	1	0	0	0	1	1/1
BKA/BKA/UEA	13	4	6	4	2	6/13
BKA/TKA/UEA	1	0	1	0	0	0/1
BKA/BKA	9	3	5	2	0	2/9
BKA/UEA	2	0	0	0	0	0/2
BKA/TKA	1	0	0	1	0	1/1
BKA/-	22	1	9	1	0	1/22
TKA/TKA/UEA	1	1	1	0	0	0/1
TKA/TKA	1	0	0	0	0	0/1
TKA/-	1	0	0	0	0	0/1
TAA/UEA	1	0	0	0	0	0/1
TAA/-	3	0	0	0	0	0/3
UEA/-	1	0	0	0	0	0/1

HipDis, hip disarticulation; AKA, above knee amputation; BKA, below knee amputation; TKA, though knee amputation, TAA, though ankle amputation; UEA, upper extremity amputation, which includes above elbow, below elbow and hand amputations.

For peer review only - http://bmjopen.bmj.com/site/about/guidelines.xhtml

Table 3: Distribution of pediatric amputations

Age	Amputation Type	Pelvic Fracture	Perineal, Gluteal, Genital Injury	Disposition
16	AKA/BKA	No	No	Survived
14	AKA/AKA	No	Yes	DOW
14	AKA/UEA/UEA	No	No	Survived
11	UEA/TKA/AKA	Yes	No	DOW
10	BKA/BKA/UEA	No	No	KIA
10	UEA/TAA	No	No	Survived
10	BKA/AKA	No	Yes	Survived
10	BKA/BKA	No	No	KIA
6	UEA	No	No	Survived

AKA, above knee amputation; BKA, below knee amputation; TKA, though knee amputation, TAA, though ankle amputation; UEA, upper extremity amputation, which includes above elbow, below elbow and hand amputations; DOW, died of wounds; KIA, killed in action.

For peer review only

Legend for figures

Figure 1: Age ranges of AP-IED targeted victims

Figure 2: Numbers of traumatic limb amputations per targeted victim of the AP-IED

Figure 3: X-ray taken for placement of a pelvic external fixator showing: disruption of pelvic ring by the force of an anti-personnel improvised explosive device; the arrow indicates combat gauze packing a severe perineal injury; silica laden soil injection by the explosion.

For peer review only

For peer review only - http://bmjopen.bmj.com/site/about/guidelines.xhtml

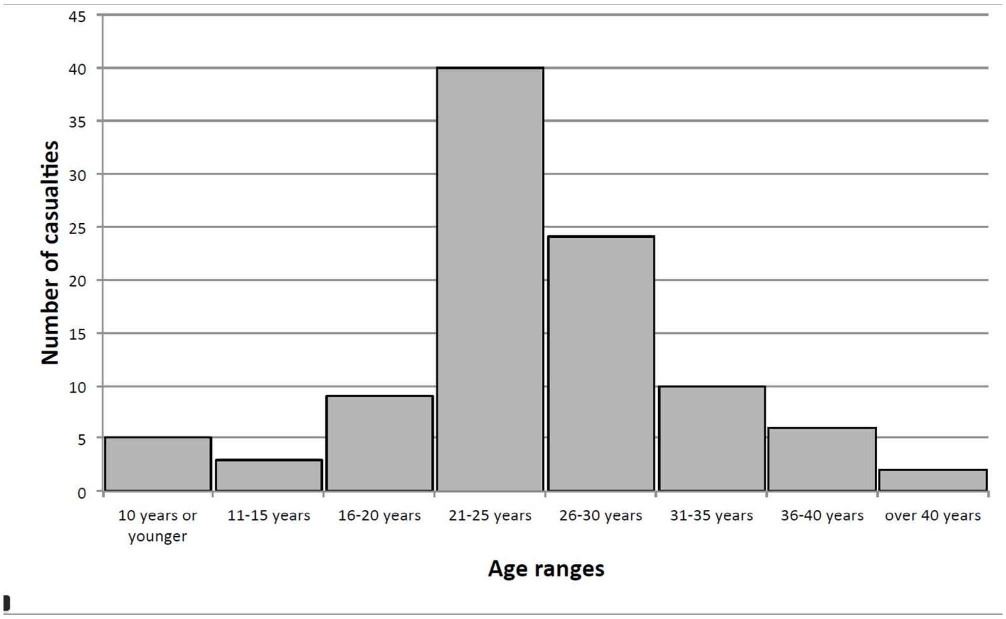

Figure 1: Age ranges of AP-IED targeted victims

125x76mm (300 x 300 DPI)

review only

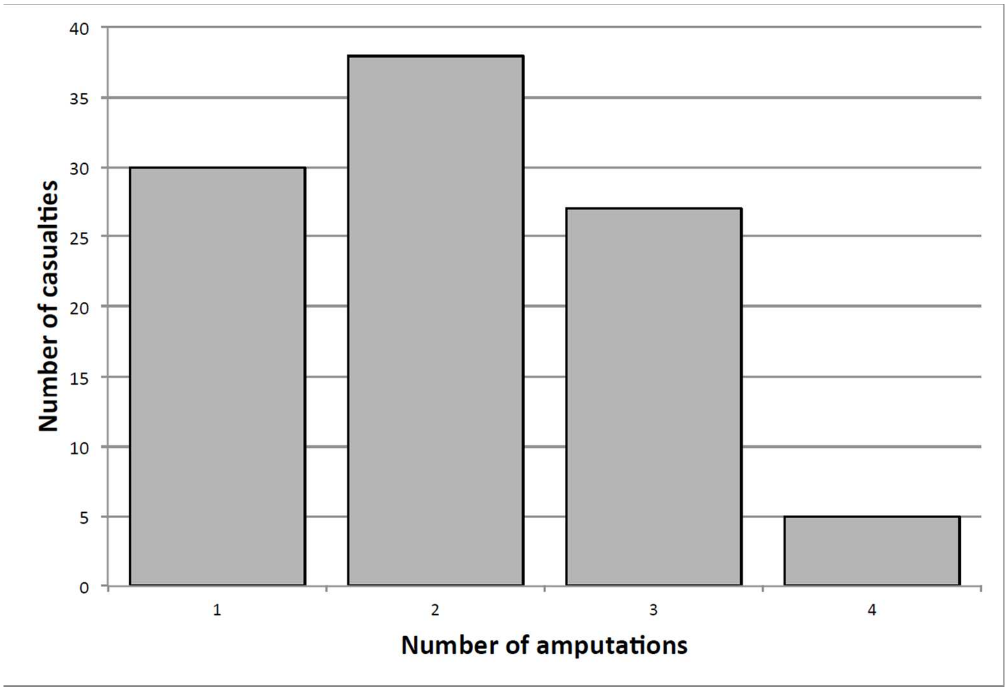

Figure 2: Numbers of traumatic limb amputations per targeted victim of the AP-IED

112x76mm (300 x 300 DPI)

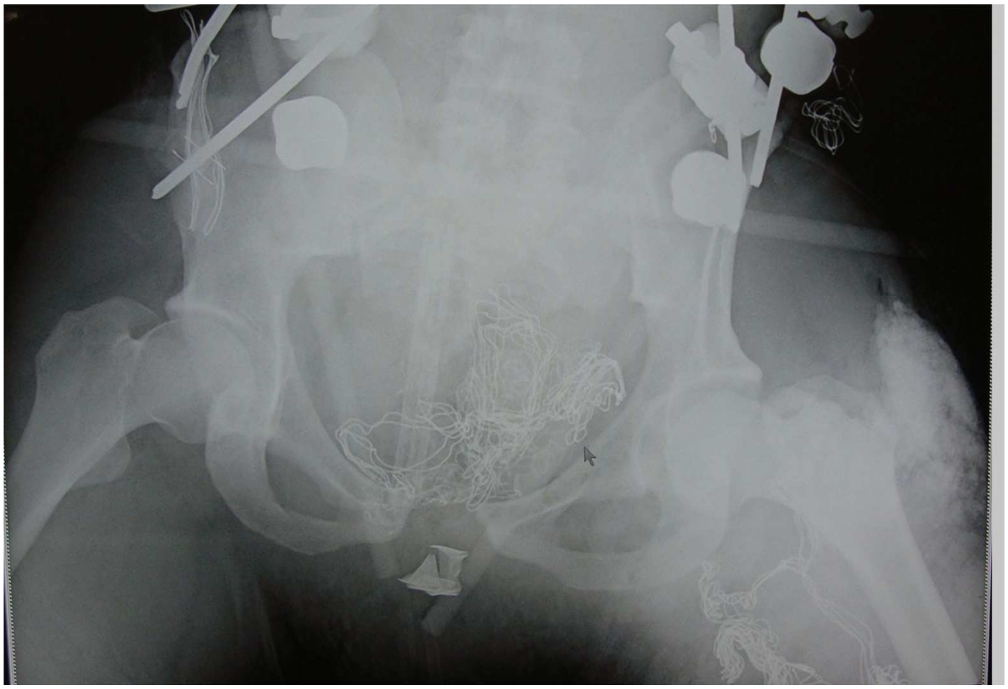

Figure 3: X-ray taken for placement of a pelvic external fixator showing: disruption of pelvic ring by the force of an anti-personnel improvised explosive device; the arrow indicates combat gauze packing a severe perineal injury; silica laden soil injection by the explosion.

440x300mm (300 x 300 DPI)

iew only

STROBE Statement—checklist of items that should be included in reports of observational studies

	Item No	Recommendation
**Title and abstract**	1	(*a*) Indicate the study's design with a commonly used term in the title or the abstract **Pg 4**
		(*b*) Provide in the abstract an informative and balanced summary of what was done and what was found **Pg 4**
**Introduction**		
Background/rationale	2	Explain the scientific background and rationale for the investigation being reported **Pg 7-8**
Objectives	3	State specific objectives, including any prespecified hypotheses **Pg 9**
**Methods**		
Study design	4	Present key elements of study design early in the paper **Pg 9**
Setting	5	Describe the setting, locations, and relevant dates, including periods of recruitment, exposure, follow-up, and data collection **Pg 9**
Participants	6	(*a*) *Cohort study*—Give the eligibility criteria, and the sources and methods of selection of participants. Describe methods of follow-up **Pg 9**<br>*Case-control study*—Give the eligibility criteria, and the sources and methods of case ascertainment and control selection. Give the rationale for the choice of cases and controls<br>*Cross-sectional study*—Give the eligibility criteria, and the sources and methods of selection of participants
		(*b*) *Cohort study*—For matched studies, give matching criteria and number of exposed and unexposed N/A<br>*Case-control study*—For matched studies, give matching criteria and the number of controls per case
Variables	7	Clearly define all outcomes, exposures, predictors, potential confounders, and effect modifiers. Give diagnostic criteria, if applicable **Pg 9**
Data sources/ measurement	8*	For each variable of interest, give sources of data and details of methods of assessment (measurement). Describe comparability of assessment methods if there is more than one group **Pg 9**
Bias	9	Describe any efforts to address potential sources of bias **Pg 10**
Study size	10	Explain how the study size was arrived at **Pg 9**
Quantitative variables	11	Explain how quantitative variables were handled in the analyses. If applicable, describe which groupings were chosen and why **N/A**
Statistical methods	12	(*a*) Describe all statistical methods, including those used to control for confounding **Pg 10**
		(*b*) Describe any methods used to examine subgroups and interactions **Pg 10**
		(*c*) Explain how missing data were addressed **Pg 10**
		(*d*) *Cohort study*—If applicable, explain how loss to follow-up was addressed<br>*Case-control study*—If applicable, explain how matching of cases and controls was addressed<br>*Cross-sectional study*—If applicable, describe analytical methods taking account of sampling strategy
		(*e*) Describe any sensitivity analyses

Continued on next page

For peer review only

For peer review only - http://bmjopen.bmj.com/site/about/guidelines.xhtml

**Results**		
Participants	13*	(a) Report numbers of individuals at each stage of study—eg numbers potentially eligible, examined for eligibility, confirmed eligible, included in the study, completing follow-up, and analysed **Pg 10**
		(b) Give reasons for non-participation at each stage
		(c) Consider use of a flow diagram
Descriptive data	14*	(a) Give characteristics of study participants (eg demographic, clinical, social) and information on exposures and potential confounders **Pg 10**
		(b) Indicate number of participants with missing data for each variable of interest **Pg 10 – Table 1**
		(c) *Cohort study*—Summarise follow-up time (eg, average and total amount)
Outcome data	15*	*Cohort study*—Report numbers of outcome events or summary measures over time
		Case-control study—Report numbers in each exposure category, or summary measures of exposure
		Cross-sectional study—Report numbers of outcome events or summary measures
Main results	16	(*a*) Give unadjusted estimates and, if applicable, confounder-adjusted estimates and their precision (eg, 95% confidence interval). Make clear which confounders were adjusted for and why they were included **Pg 10-11**
		(*b*) Report category boundaries when continuous variables were categorized
		(*c*) If relevant, consider translating estimates of relative risk into absolute risk for a meaningful time period
Other analyses	17	Report other analyses done—eg analyses of subgroups and interactions, and sensitivity analyses
**Discussion**		
Key results	18	Summarise key results with reference to study objectives **Pg 12**
Limitations	19	Discuss limitations of the study, taking into account sources of potential bias or imprecision. Discuss both direction and magnitude of any potential bias **Pg 12**
Interpretation	20	Give a cautious overall interpretation of results considering objectives, limitations, multiplicity of analyses, results from similar studies, and other relevant evidence **Pg 13-14**
Generalisability	21	Discuss the generalisability (external validity) of the study results **Pg 15**
**Other information**		
Funding	22	Give the source of funding and the role of the funders for the present study and, if applicable, for the original study on which the present article is based **Pg 3**

*Give information separately for cases and controls in case-control studies and, if applicable, for exposed and unexposed groups in cohort and cross-sectional studies.

Note: An Explanation and Elaboration article discusses each checklist item and gives methodological background and published examples of transparent reporting. The STROBE checklist is best used in conjunction with this article (freely available on the Web sites of PLoS Medicine at http://www.plosmedicine.org/, Annals of Internal Medicine at http://www.annals.org/, and Epidemiology at http://www.epidem.com/). Information on the STROBE Initiative is available at www.strobe-statement.org.

For peer review only - http://bmjopen.bmj.com/site/about/guidelines.xhtml
